# Incidence and prevalence of musculoskeletal health conditions in survivors of childhood and adolescent cancers: A report from the Swiss childhood cancer survivor study

**DOI:** 10.1002/cam4.7204

**Published:** 2024-04-23

**Authors:** Salome Christen, Katharina Roser, Luzius Mader, Maria Otth, Katrin Scheinemann, Grit Sommer, Claudia Kuehni, Gisela Michel

**Affiliations:** ^1^ Faculty of Health Sciences and Medicine University of Lucerne Lucerne Switzerland; ^2^ Cancer Registry Bern Solothurn University of Bern Bern Switzerland; ^3^ Department of Oncology University Children's Hospital Zurich Zurich Switzerland; ^4^ Division of Pediatric Hematology/Oncology Children's Hospital of Eastern Switzerland St. Gallen Switzerland; ^5^ Department of Pediatrics McMaster Children's Hospital and McMaster University Hamilton Ontario Canada; ^6^ Swiss Childhood Cancer Registry Institute of Social and Preventive Medicine, University of Bern Bern Switzerland; ^7^ Division of Pediatric Hematology/Oncology, Department of Pediatrics Inselspital, Bern University Hospital, University of Bern Bern Switzerland

**Keywords:** childhood cancer, musculoskeletal health conditions, registry, scoliosis, survivors, Switzerland

## Abstract

**Purpose:**

Childhood cancer and its treatment can cause damage to the musculoskeletal system. We aimed to determine the incidence and prevalence of musculoskeletal health conditions (MSHC) in survivors, and to investigate differences by cancer‐related characteristics.

**Methods:**

We used data from the Childhood Cancer Registry and the Swiss Childhood Cancer Survivor Study, including survivors (≥5 years since diagnosis; diagnosed 1976–2015 at <20 years of age) aged ≥15 years at study. Cumulative incidence and prevalence of MSHCs (osteoporosis, limb length discrepancy, limited joint mobility, bone/joint pain, scoliosis, changes to chest/ribs and amputation) were calculated from self‐reported data.

**Results:**

We included 2645 survivors (53% men; median age 24 years, range 15–59 years). Prevalence and cumulative incidence of *any MSHC* was 21% and 26%, respectively. Incidence rate for *any MSHC* was 15.6/1000 person‐years. Scoliosis (8%), bone/joint pain (7%) and limited joint mobility (7%) were the most prevalent MSHC. MSHC co‐occurred with other health conditions in 87% of survivors. We found increased rates of MSHC in women (RR = 1.4, 95%CI: 1.2–1.7), bone tumour survivors (RR = 6.0, 95%CI: 4.5–7.9), survivors older at diagnosis (11–15 years: RR = 1.8, 95%CI: 1.5–2.3), after a relapse (RR = 1.5, 95%CI: 1.3–1.9), treatment with surgery (RR = 1.2, 95%CI: 1.0–1.5), chemotherapy (RR = 1.4, 95%CI: 1.1–1.8) or stem cell transplantation (RR = 1.6, 95%CI: 1.0–2.5), and more recent year of diagnosis (2011–2015: RR = 4.3, 95%CI: 2.8–6.8).

**Conclusion:**

MSHCs are prevalent in survivors, the risk is increasing in younger survivor cohorts, and MSHCs usually occur in multimorbid survivors. Strengthening of rehabilitation services and appropriate referrals are needed to mitigate the effects of the cancer and cancer treatment.

## 1 INTRODUCTION

Damage to the musculoskeletal system by childhood cancer itself or its treatment can often not be prevented without jeopardising the patient's survival.^1^ There is a wide range of musculoskeletal health conditions (MSHCs) that can develop in childhood and adolescent cancer survivors (CCS), including low bone mineral density and osteoporosis, spinal deformities such as scoliosis or kyphosis, muscle weakness, arm‐ or leg‐length discrepancies, or amputation.^1^, ^2^, ^3^, ^4^, ^5^ With improved survival rates, nowadays exceeding 80% in high‐income countries, cancer‐ and treatment‐related health conditions pose a considerable problem for childhood cancer survivors.^6^

In the general population, MSHCs are among the leading contributors to years lived with disability,^7^ and, consequently, the leading contributor to the need for rehabilitation.^8^ Compared to the general population or siblings, MSHCs are more prevalent in CCS^9^, ^10^, ^11^, ^12^: 10.4% of survivors in the in the Childhood Cancer Survivor Study experience MSHC compared to 1.4% of siblings (risk ratio 8.5 (95%CI: 6.5–11.2)).^12^ Data from the St. Jude Lifetime Cohort Study show that MSHCs are the third most common health problem in long‐term CCS, with a cumulative incidence of 83.6% at age 50 years.^13^ Additionally, MSHCs are associated with low health‐related quality of life in CCS,^9^ and are among the late effects that have the strongest negative impact on physical functioning.^9^, ^12^ MSHCs can also cause secondary complications, such as back pain, hip pain, fractures, muscular imbalances, gait abnormalities, or impaired mobility.^4^, ^14^, ^15^ Fortunately, many MSHCs are amenable to rehabilitation, and early intervention can help to prevent or ameliorate impaired physical functioning or disability.^16^

Prevalence of MSHC in cohorts of CCS varies from 2% to 62% depending on the definition of MSHCs and the average age of the cohort.^11^, ^12^, ^17^, ^18^, ^19^, ^20^ Incidence of MSHCs is largest in the first years after diagnosis, but evidence suggests that MSHCs can also develop decades after diagnosis,^10^ although there are differences by primary cancer diagnosis.^21^ All treatment modalities used for treating children and adolescents with cancer can cause MSHCs.^1^, ^3^ MSHCs seem to become more frequent with current treatment protocols, but this temporal trend has only been analysed in a subgroup of survivors of acute lymphoblastic leukaemia.^19^ There is also an indication for age‐ and sex‐specific differences in MSHC after childhood cancer.^10^, ^11^

Most studies analysing MSHCs have merged them to a single outcome, disregarding distinct entities.^11^, ^13^, ^17^, ^18^ Only few studies have presented results stratified for specific MSHCs.^10^, ^12^ Other studies have determined incidence or prevalence of specific MSHCs, but in selected diagnostic groups only.^19^, ^20^, ^22^, ^23^ We used data from a nationwide, population‐based cohort study, the Swiss Childhood Cancer Survivor Study (SCCSS), (1) to determine the cumulative incidence, incidence rates, and prevalence of any and specific MSHCs (osteoporosis, arm‐ or leg‐length discrepancy, limited joint mobility, persistent pain in bones or joints, scoliosis, amputation), and (2) to evaluate differences in incidence rates of any and the specific MSHCs by sex, age, type of cancer diagnosis, age at diagnosis, type of treatment and year of diagnosis.

## 2 METHODS

### 2.1 Sample and procedure

#### 2.1.1 Survivor population

The Childhood Cancer Registry (ChCR) is a nationwide, population‐based cancer registry for all Swiss residents diagnosed <20 years of age with leukaemia, lymphoma, central nervous system (CNS) tumours, malignant solid tumours, or Langerhans cell histiocytosis.^24^ The SCCSS is a nationwide, population‐based, prospective cohort study including all children and adolescents registered in the ChCR, diagnosed with cancer since 1976 at an age of <20 years, who survived at least 5 years.^25^ For the current analysis, we included survivors aged ≥15 years at study, because information on MSHC was assessed differently in the questionnaire used in younger survivors.

#### 2.1.2 Setting

Survivors first received a letter with study information and the option to refuse participation from their former treatment clinic. Two weeks later, the SCCSS research team at the University of Bern sent survivors a paper‐based questionnaire with a pre‐paid return envelope. If they did not respond, they received a reminder letter with another questionnaire 4–6 weeks later, and then survivors were contacted by phone for a second reminder. The SCCSS questionnaire assessed information on health conditions, health service utilisation, health behaviours and socio‐demographic characteristics. Data were collected from 2007 to 2022 and the overall response rate was 56%. Informed consent was obtained from all participating survivors. A detailed description of the SCCSS is provided elsewhere.^25^

Ethical approval of the SCCSS was granted by the Ethics Committee of the Canton of Bern (KEK‐No. 166/14 and 2021–01462).

### 2.2 Measurements

#### 2.2.1 Musculoskeletal health conditions

The following MSHCs were assessed through self‐report in the SCCSS questionnaire: osteoporosis, arm‐ or leg length discrepancy, limited joint mobility, persistent pain in bones or joints, scoliosis, changes to the chest and/or ribs. For each MSHC, participants were asked whether, at any time in their life, they had ever had this health condition (*incidence*: yes/no). If yes, they were asked to indicate since when they had this health condition (*incidence year*) and whether they were currently still experiencing it (*prevalence*: yes/no). Participants who answered ‘no’ or who left a question blank were included in the ‘no’ categories. If participants did not complete any question on health conditions, they were coded to have missing information. We additionally generated an overall variable (*any MSHC*) and coded ‘yes’ if participants experienced at least one of the MSHCs.

Information on musculoskeletal surgeries was coded from open answers to the question whether the participant had had any surgeries during or after their primary cancer treatment into amputation, rotationplasty, joint replacement or arthrodesis, limb lengthening/shortening, scoliosis surgery or spondylodesis, thorax surgery, surgery due to a fracture, or any other musculoskeletal surgery. We additionally generated an overall variable (*any musculoskeletal surgery*) and coded ‘yes’ if participants had had at least one of the above musculoskeletal surgeries.

#### 2.2.2 Explanatory variables

We obtained prospectively collected medical information from the ChCR: sex (male, female), cancer diagnosis (coded according to the International Classification of Childhood Cancer‐3^26^), surgery (yes/no), chemotherapy (yes/no), radiotherapy (yes/no), and stem cell transplantation (yes/no), year of diagnosis (1976–2015, 5‐year increments), age at diagnosis (years), and relapse or second malignancy (yes/no). Age at study (years) was assessed in the SCCSS questionnaire. We calculated the sum of organ systems affected by health conditions as indicated by the participant (organ systems: neurological, musculoskeletal, heart/circulatory, vision, hearing, hormonal, respiratory, digestive, urinary, cancer‐related fatigue and mental health).

### 2.3 Data analysis

For all analyses, we used Stata version 17.0.^27^ All tests were two‐sided and considered statistically significant if *p* < 0.05. Analyses are based on a completely anonymised dataset. We generated time since diagnosis (years), age at incidence (years), time since diagnosis at incidence (years), and categorical variables with a priori defined categories for: age at diagnosis (0–5 years, 6–10 years, 11–15 years and 16–20 years), time since diagnosis (5–50 years, 5‐year increments), age at study (15–64 years, 10‐year increments).

We used descriptive statistics to describe the prevalence, cumulative incidence, and incidence rate for *any MSHC* and each specific MSHC. We computed cumulative incidence and point prevalence for *any MSHC* and the specific MSHCs. We calculated the proportion of survivors for whom the condition is no longer prevalent. We computed incidence rates for any and the specific MSHCs by calculating the number of participants who answered *incidence* with ‘yes’ and divided by total observation time at risk. The starting point for time at risk was the age at diagnosis, the endpoint was the age at incidence, or the age at study for survivors who did not report a MSHC. We calculated incidence rates for any and specific MSHCs stratified by sex, age, cancer diagnosis, age at diagnosis, treatment, and year of diagnosis. We calculated rate ratios to compare different groups.

We calculated the missing values of the primary outcomes (Table S1). We present complete case analyses, as the performed sensitivity analyses showed that the results remained stable with different imputation scenarios. The complete case analysis provided the most conservative results (a detailed description and results of the sensitivity analyses are presented in Tables S4A–S6C).

## 3 RESULTS

Overall, 2645 survivors (53% male) were included. Participating survivors had most often been diagnosed with leukaemia (29%), lymphoma (22%) and CNS tumours (16%) at a median age of 10 years (IQR 10.0). Median age at study was 24 years (IQR 10.2), and a median of 16 years (IQR 11.6) had passed since their cancer diagnosis (Table 1).

**TABLE 1:** Characteristics of the study population (*N* = 2645).

	*N*	%
Sex		
Male	1401	53.0
Female	1244	47.0
Missing	0	0.0
Age at study, years, median (IQR; range)	24.0 (10.20; 15–59)	
15–24	1436	54.3
25–34	836	31.6
35–44	295	11.2
45–54	73	2.8
55–64	3	0.1
Missing	2	0.1
Diagnosis		
Leukaemia	756	28.6
Lymphoma	577	21.8
CNS tumour	417	15.8
Neuroblastoma	87	3.3
Retinoblastoma	45	1.7
Renal tumour	121	4.6
Hepatic tumour	15	0.6
Malignant bone tumour	139	5.3
Soft tissue sarcoma	166	6.3
Germ cell tumour	151	5.7
Other tumour	81	3.1
Langerhans cell histiocytosis	90	3.4
Missing	0	0.0
Treatment^a^		
Surgery	1757	66.4
Chemotherapy	1988	75.2
Radiotherapy	859	32.5
Stem cell therapy	91	3.4
Relapse		
Yes	2090	79.0
No	555	21.0
Missing	0	0.0
No. of organ systems affected by late effects		
None	652	24.7
1–2	1134	42.9
3–4	490	18.5
5+	240	9.1
Missing	129	4.9
Age at diagnosis, years, median (IQR; range)	10.0 (10.00; 0–20)	
0–5	818	30.9
6–10	598	22.6
11–15	815	30.8
16–20	414	15.7
Missing	0	0.0
Time since diagnosis, years, median, (IQR; range)	15.6 (11.62; 5–42)	
5–9	655	24.8
10–14	583	22.0
15–19	584	22.1
20–24	434	16.4
25–29	252	9.5
30–34	106	4.0
35–39	27	1.0
40–44	2	0.1
Missing	2	0.1
Year of diagnosis		
1976–1980	189	7.1
1981–1985	315	11.9
1986–1990	428	16.2
1991–1995	535	20.2
1996–2000	431	16.3
2001–2005	316	11.9
2006–2010	217	8.2
2011–2015	214	8.1
Missing	0	0.0

Abbreviations: CNS, central nervous system; IQR, interquartile range.

^a^ Surgery: *n* = 93 missings; chemotherapy: *n* = 107 missings; radiotherapy: *n* = 130 missings; stem cell therapy: *n* = 150 missings.

Prevalence of *any MSHC* at the time of study participation was 21% (*n* = 533; Table 2). The most frequent MSHCs were scoliosis (*n* = 207, 8%), persistent pain in bones or joints (*n* = 188, 7%) and limited joint mobility (*n* = 170, 7%). At the time of study, most survivors with *any MSHC* had one MSHC (*n* = 373, 70%), 19% (*n* = 100) had two, 8% (*n* = 42) had three, and a minority had four or five MSHCs (*n* = 18, 3%; Table 2). Of survivors with *any MSHC*, only 13% (*n* = 67) had no other health condition, whereas 87% (*n* = 466) also had at least another health condition in another organ system.

**TABLE 2:** Prevalence and cumulative incidence of musculoskeletal health conditions and musculoskeletal surgeries in adolescent and adult 5‐year survivors of childhood and adolescent cancer (total sample size: *N* = 2645).

	Prevalence	Cumulative incidence	Condition no longer prevalent	Age at incidence in years	Time since diagnosis at incidence^a^ in years
	*n* (%)	*n* (%)	*n* ^b^ (%^c^)	Median (IQR; range)	Median (IQR; range)
Any musculoskeletal health condition	533 (21.2)	643 (26.2)	154 (24.0)	16 (8; 0–56)	5 (11; 0–38)
No. of musculoskeletal health conditions^d^					
1	373 (70.0)	n.a.	n.a.	n.a.	n.a.
2	100 (18.8)	n.a.	n.a.	n.a.	n.a.
3	42 (7.9)	n.a.	n.a.	n.a.	n.a.
4–5	18 (3.4)	n.a.	n.a.	n.a.	n.a.
Osteoporosis	61 (2.4)	103 (4.1)	42 (40.8)	17 (10; 2–45)	5 (12; 0–30)
Arm‐ or leg‐length discrepancy	107 (4.3)	121 (4.8)	23 (19.0)	13 (10; 0–43)	2 (7; 0–32)
Limited joint mobility	170 (6.8)	225 (8.9)	60 (26.7)	15 (7; 0–43)	2 (7; 0–32)
Persistent pain in bones or joints	188 (7.5)	280 (11.1)	93 (33.2)	18 (9; 1–56)	9 (14; 0–38)
Scoliosis	207 (8.2)	230 (9.1)	49 (21.3)	14 (7; 0–44)	6 (9; 0–29)
Changes to chest/ribs	41 (1.6)	50 (2.0)	13 (26.0)	15 (10; 6–50)	3 (8; 0–33)
Any MSK surgery	n.a.	380 (15.5)	n.a.	n.a.	n.a.
No. of MSK surgeries^e^					
1	n.a.	262 (68.9)	n.a.	n.a.	n.a.
2	n.a.	90 (23.7)	n.a.	n.a.	n.a.
3	n.a.	22 (5.8)	n.a.	n.a.	n.a.
4	n.a.	4 (1.1)	n.a.	n.a.	n.a.
Amputation	n.a.	42 (1.7)	n.a.	n.a.	n.a.
Rotationplasty	n.a.	6 (0.2)	n.a.	n.a.	n.a.
Joint replacement or arthrodesis	n.a.	24 (1.0)	n.a.	n.a.	n.a.
Limb lengthening/shortening	n.a.	13 (0.5)	n.a.	n.a.	n.a.
Scoliosis surgery or spondylodesis	n.a.	10 (0.4)	n.a.	n.a.	n.a.
Thorax surgery	n.a.	11 (0.5)	n.a.	n.a.	n.a.
Fracture surgery	n.a.	99 (4.1)	n.a.	n.a.	n.a.
Any other MSK surgery	n.a.	153 (6.3)	n.a.	n.a.	n.a.

*Note*: Proportions were calculated excluding missing values.

Abbreviations: IQR, interquartile range, MSK, musculoskeletal, n.a., not applicable.

^a^ Figure S1 displays years since diagnosis at incidence for the specific musculoskeletal health condition.

^b^ Number of survivors that indicated cumulative incidence ‘yes’ and prevalence ‘no’.

^c^ Number of survivors that indicated cumulative incidence ‘yes’ and prevalence ‘no’ divided by number of survivors that indicated cumulative incidence ‘yes’.

^d^ In survivors with a musculoskeletal health condition (*N* = 533 (100%)).

^e^ In survivors with a musculoskeletal surgery (*N* = 380 (100%)).

Cumulative incidence of *any MSHC* was 26% (*n* = 643). Cumulative incidence was highest for persistent pain in bones or joints (*n* = 280, 11%), scoliosis (*n* = 230, 9%) and limited joint mobility (*n* = 225, 9%).

Survivors were on average 16 years old (IQR 8) at incidence of the first MSHC, but age at incidence varied considerably (range 0–56 years at incidence). Most MSHCs manifested within the first 10 years after diagnosis (*any MSHC*: median 5 years (IQR 11); Table 2, Figure S1). One fourth (24%) of survivors who experienced a MSHC since their cancer diagnosis reported that this MSHC was currently no longer prevalent (*n* = 154, 24%; Table 2). We found that 16% of survivors had had a musculoskeletal surgery: most frequent were surgeries due to a fracture (*n* = 99, 4%), amputation (*n* = 42, 2%) and joint replacement or arthrodesis (*n* = 24, 1%; Table 2).

**FIGURE 1:**
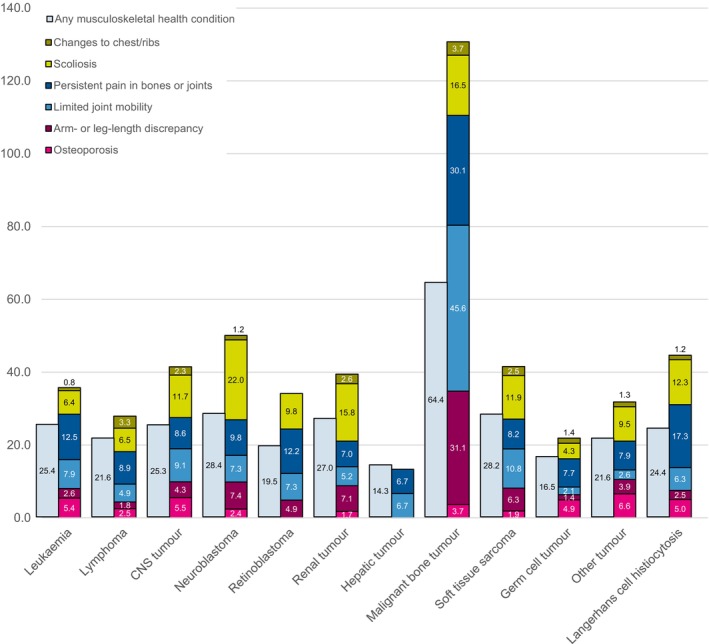
The cumulative incidence of specific musculoskeletal health conditions stratified by diagnosis. The cumulative incidence (%) of musculoskeletal health conditions is presented; numbers can exceed 100% for the specific conditions, because survivors can experience more than one of the conditions simultaneously.

MSHCs were most frequent in the survivors of malignant bone tumours (cumulative incidence of any condition: 64%), neuroblastoma (28%) and soft tissue sarcoma (28%; Figure 1). The cumulative incidence of the specific MSHC stratified by additional cancer‐related characteristics is presented in Table S2. Compared to survivors of leukaemia, survivors of malignant bone tumours had higher rates of *any MSHC* (rate ratio (RR) = 6.0, 95%CI: 4.5–7.9; Table 3). We found that female survivors had higher rates of *any MSHC* (RR = 1.4, 95%CI: 1.2–1.7) as compared to male survivors. Having had a relapse was associated with higher rates of any MSHC (RR = 1.5, 95%CI: 1.3–1.9). Compared to survivors aged 0–5 years at diagnosis, we found that survivors aged 6–10 years and 11–15 years at diagnosis had higher rates of *any MSHC* (6–10 years: RR = 1.5, 95%CI: 1.2–1.9; 11–15 years: RR = 1.8, 95%CI: 1.5–2.3). When stratifying these results by diagnosis, we found that older age at diagnosis remained associated with higher rates of any MSHC for most diagnoses (Tables S3 and S4). Surgery (RR = 1.2, 95%CI: 1.0–1.5), chemotherapy (RR = 1.4, 95%CI: 1.1–1.8) and stem cell therapy (RR = 1.6, 95%CI: 1.0–2.5) were all independently associated with higher rates of *any MSHC* (Table 3).

**TABLE 3:** Incidence rates (per 1000 person‐years) and rate ratios of musculoskeletal health conditions in adolescent and adult 5‐year survivors of childhood and adolescent cancer (unadjusted; total sample size: *N* = 2645).

	Any musculoskeletal health condition			Osteoporosis			Arm‐ or leg‐length discrepancy			Limited joint mobility			Persistent pain in bones or joints			Scoliosis			Changes to chest/ribs		
	Incidence rate	Rate ratios (95% CI)	*p*‐value	Incidence rate	Rate ratios (95% CI)	*p*‐value	Incidence rate	Rate ratios (95% CI)	*p*‐value	Incidence rate	Rate ratios (95% CI)	*p*‐value	Incidence rate	Rate ratios (95% CI)	*p*‐value	Incidence rate	Rate ratios (95% CI)	*p*‐value	Incidence rate	Rate ratios (95% CI)	*p*‐value
Overall	15.6 (14.3–17.0)	n.a.		2.1 (1.7–2.6)	n.a.		2.2 (1.8–2.8)	n.a.		4.9 (4.2–5.6)	n.a.		5.9 (5.2–6.8)	n.a.		4.2 (3.6–4.9)	n.a.		0.9 (0.6–1.2)	n.a.	
Sex																					
Males	13.2	Ref.		1.5	Ref.		1.9	Ref.		4.4	Ref.		4.9	Ref.		3.9	Ref.		0.9	Ref.	
Females	18.4	**1.4 (1.2–1.7)**	**<0.001**	2.8	**1.9 (1.2–3.1)**	**0.003**	2.6	1.3 (0.9–2.1)	0.164	5.4	1.2 (0.9–1.6)	0.179	7.1	**1.4 (1.1–1.9)**	**0.006**	4.5	1.2 (0.8–1.6)	0.366	0.8	0.9 (0.4–1.8)	0.704
Diagnosis																					
Leukaemia	14.2	Ref.		2.8	Ref.		0.8	Ref.		4.4	Ref.		6.8	Ref.		2.4	Ref.		0.3	Ref.	
Lymphoma	12.4	0.9 (0.7–1.1)	0.299	1.5	0.6 (0.3–1.1)	0.066	0.8	1.1 (0.3–3.3)	0.839	2.3	**0.5 (0.3–0.9)**	**0.012**	5.0	0.7 (0.5–1.1)	0.110	2.5	1.0 (0.6–1.9)	0.873	1.5	**4.4 (1.3–18.9)**	**0.006**
CNS tumour	15.3	1.1 (0.8–1.4)	0.616	2.6	0.9 (0.5–1.8)	0.860	1.8	2.3 (0.8–6.4)	0.073	5.0	1.1 (0.7–1.8)	0.605	4.4	**0.6 (0.4–1.0)**	**0.048**	6.1	**2.5 (1.5–4.3)**	**<0.001**	1.2	**3.6 (0.9–16.9)**	**0.042**
Neuroblastoma	14.4	1.0 (0.6–1.6)	0.939	1.2	0.4 (0.1–1.7)	0.250	2.6	3.4 (0.8–12.2)	0.066	3.2	0.7 (0.2–1.8)	0.510	4.6	0.7 (0.3–1.4)	0.307	9.9	**4.1 (2.0–8.0)**	**<0.001**	0.0	0.0 (0.0–11.3)	0.605
Retinoblastoma	9.2	0.6 (0.3–1.4)	0.260	0.0	0.0 (0.0–1.7)	0.102	2.4	3.2 (0.3–15.3)	0.191	3.7	0.8 (0.2–2.6)	0.844	5.0	0.7 (0.2–2.0)	0.592	3.7	1.5 (0.3–5.0)	0.471	0.0	0.0 (0.0–21.8)	0.764
Renal tumour	14.3	1.0 (0.6–1.6)	0.970	0.9	0.3 (0.0–1.3)	0.107	2.5	**3.4 (0.9–11.2)**	**0.047**	2.4	0.6 (0.2–1.4)	0.199	2.9	**0.4 (0.2–1.0)**	**0.028**	7.0	**2.9 (1.4–5.7)**	**0.004**	0.5	1.5 (0.0–14.8)	0.704
Hepatic tumour	8.9	0.6 (0.1–2.3)	0.561	0.0	0.0 (0.0–5.4)	0.487	0.0	0.0 (0.0–23.1)	0.822	3.9	0.9 (0.0–5.1)	0.998	3.9	0.6 (0.0–3.3)	0.657	0.0	0.0 (0.0–7.0)	0.572	0.0	0.0 (0.0–70.7)	0.919
Malignant bone tumour	84.8	**6.0 (4.5–7.9)**	**<0.001**	2.5	0.9 (0.3–2.4)	0.900	27.4	**36.2 (17.3–84.8)**	**<0.001**	43.1	**9.8 (6.6–14.6)**	**<0.001**	21.3	**3.1 (2.0–4.7)**	**<0.001**	11.9	**4.9 (2.6–9.0)**	**<0.001**	2.6	**7.8 (1.7–39.5)**	**0.004**
Soft tissue sarcoma	17.2	1.2 (0.8–1.8)	0.302	0.4	**0.1 (0.0–0.8)**	**0.010**	3.2	**4.2 (1.4–12.3)**	**0.006**	6.2	1.4 (0.7–2.5)	0.251	3.9	0.6 (0.3–1.1)	0.085	4.9	2.0 (0.9–4.1)	0.053	1.5	**4.6 (0.9–24.8)**	**0.044**
Germ cell tumour	9.8	0.7 (0.4–1.1)	0.111	3.1	1.1 (0.4–2.6)	0.762	0.4	0.6 (0.0–4.1)	0.672	0.9	**0.2 (0.0–0.8)**	**0.006**	4.0	0.6 (0.3–1.2)	0.116	1.8	0.7 (0.2–2.1)	0.617	0.4	1.3 (0.0–13.3)	0.765
Other tumour	11.4	0.8 (0.4–1.5)	0.518	2.9	1.0 (0.2–3.3)	0.882	0.9	1.2 (0.0–8.8)	0.781	0.9	0.2 (0.0–1.2)	0.069	3.9	0.6 (0.2–1.5)	0.270	2.0	0.8 (0.1–3.3)	0.875	0.9	2.8 (0.1–28.1)	0.407
Langerhans cell histiocytosis	12.9	0.9 (0.5–1.6)	0.753	2.3	0.8 (0.2–2.6)	0.794	0.8	1.0 (0.0–7.3)	0.909	3.0	0.7 (0.2–1.9)	0.498	8.7	1.3 (0.6–2.4)	0.442	7.0	**2.9 (1.2–6.3)**	**0.012**	0.7	2.3 (0.0–22.9)	0.488
Relapse																					
No	14.1	Ref.		1.8	Ref.		2.2	Ref.		4.2	Ref.		5.6	Ref.		3.7	Ref.		0.8	Ref.	
Yes	21.7	**1.5 (1.3–1.9)**	**<0.001**	3.2	**1.8 (1.1–2.9)**	**0.020**	2.5	1.1 (0.7–1.9)	0.589	7.5	**1.8 (1.3–2.4)**	**0.001**	7.5	1.4 (1.0–1.8)	0.051	6.0	**1.6 (1.1–2.3)**	**0.008**	1.2	1.5 (0.6–3.2)	0.280
Age at diagnosis (years at dx)																					
0–5	11.7	Ref.		1.0	Ref.		1.5	Ref.		3.0	Ref.		4.1	Ref.		4.8	Ref.		0.7	Ref.	
6–10	17.3	**1.5 (1.2–1.9)**	**0.001**	1.9	1.9 (0.9–4.0)	0.080	3.7	**2.4 (1.4–4.3)**	**0.002**	5.6	**1.8 (1.2–2.8)**	**0.004**	5.4	1.3 (0.9–1.9)	0.175	4.7	1.0 (0.6–1.5)	0.890	0.3	0.4 (0.1–1.7)	0.217
11–15	21.5	**1.8 (1.5–2.3)**	**<0.001**	3.5	**3.4 (1.8–6.8)**	**<0.001**	2.4	1.6 (0.8–2.9)	0.134	8.4	**2.8 (1.9–4.1)**	**<0.001**	8.8	**2.1 (1.5–3.0)**	**<0.001**	4.7	1.0 (0.6–1.4)	0.871	1.2	1.6 (0.6–4.0)	0.266
16–20	13.4	1.1 (0.9–1.5)	0.340	2.7	**2.7 (1.3–5.7)**	**0.006**	1.6	1.0 (0.4–2.3)	0.932	2.8	0.9 (0.5–1.6)	0.792	6.6	**1.6 (1.0–2.4)**	**0.024**	1.2	**0.2 (0.1–0.5)**	**<0.001**	1.6	2.1 (0.8–5.5)	0.092
Surgery																					
No	13.7	Ref.		2.8	Ref.		0.6	Ref.		3.8	Ref.		6.5	Ref.		2.6	Ref.		0.4	Ref.	
Yes	17.0	**1.2 (1.0–1.5)**	**0.022**	1.9	0.7 (0.4–1.1)	0.084	3.1	**4.9 (2.3–11.6)**	**<0.001**	5.7	**1.5 (1.1–2.1)**	**0.013**	5.8	0.9 (0.7–1.2)	0.397	5.1	**2.0 (1.3–3.1)**	**<0.001**	1.0	**2.7 (1.0–8.9)**	**0.030**
Chemotherapy																					
No	12.2	Ref.		1.1	Ref.		1.4	Ref.		3.5	Ref.		4.2	Ref.		5.4	Ref.		0.6	Ref.	
Yes	17.0	**1.4 (1.1–1.8)**	**0.004**	2.5	**2.1 (1.1–4.9)**	**0.020**	2.5	1.8 (0.9–3.8)	0.058	5.5	**1.6 (1.0–2.5)**	**0.021**	6.5	**1.6 (1.1–2.4)**	**0.014**	4.0	0.7 (0.5–1.1)	0.110	0.9	1.5 (0.6–4.9)	0.438
Radiotherapy																					
No	16.2	Ref.		2.3	Ref.		2.3	Ref.		5.3	Ref.		6.4	Ref.		3.9	Ref.		0.7	Ref.	
Yes	15.3	0.9 (0.8–1.1)	0.559	2.1	0.9 (0.6–1.5)	0.763	2.1	0.9 (0.5–1.5)	0.670	4.4	0.8 (0.6–1.2)	0.273	5.3	0.8 (0.6–1.1)	0.179	5.1	1.3 (0.9–1.8)	0.109	1.1	1.6 (0.8–3.5)	0.169
SCT																					
No	15.7	Ref.		2.1	Ref.		2.3	Ref.		4.9	Ref.		5.9	Ref.		4.3	Ref.		0.8	Ref.	
Yes	25.8	**1.6 (1.0–2.5)**	**0.026**	4.4	2.1 (0.7–5.0)	0.146	2.6	1.1 (0.2–3.5)	0.758	9.0	1.8 (0.9–3.5)	0.080	8.1	1.4 (0.6–2.7)	0.352	6.6	1.5 (0.6–3.2)	0.280	3.5	**4.6 (1.2–13.0)**	**0.018**
Year of diagnosis																					
1976–1980	8.9	Ref.		1.1	Ref.		1.2	Ref.		2.0	Ref.		3.2	Ref.		3.6	Ref.		0.8	Ref.	
1981–1985	9.3	1.1 (0.7–1.6)	0.803	1.1	1.0 (0.3–3.5)	0.978	1.3	1.1 (0.4–3.9)	0.831	2.5	1.3 (0.5–3.1)	0.587	3.8	1.2 (0.6–2.3)	0.598	3.1	0.9 (0.4–1.7)	0.645	0.3	0.4 (0.0–2.6)	0.267
1986–1990	13.4	**1.5 (1.0–2.3)**	**0.028**	1.9	1.7 (0.6–5.2)	0.305	3.3	**2.9 (1.2–8.5)**	**0.012**	4.1	**2.1 (1.0–4.7)**	**0.038**	4.8	1.5 (0.8–2.8)	0.170	4.8	1.3 (0.7–2.5)	0.335	0.6	0.8 (0.2–4.0)	0.743
1991–1995	12.9	1.5 (1.0–2.2)	0.050	1.7	1.5 (0.6–4.9)	0.393	1.4	1.2 (0.4–3.9)	0.763	3.4	1.7 (0.8–4.0)	0.139	4.8	1.5 (0.8–2.8)	0.179	3.8	1.1 (0.6–2.1)	0.839	0.6	0.8 (0.2–4.1)	0.748
1996–2000	21.3	**2.4 (1.6–3.6)**	**<0.001**	2.8	2.5 (0.9–7.8)	0.061	3.6	**3.1 (1.2–9.5)**	**0.012**	7.4	**3.7 (1.8–8.5)**	**<0.001**	8.3	**2.6 (1.4–4.8)**	**0.001**	4.6	1.3 (0.7–2.6)	0.447	1.4	1.8 (0.5–8.4)	0.360
2001–2005	23.0	**2.6 (1.7–4.0)**	**<0.001**	3.6	**3.2 (1.0–10.7)**	**0.025**	2.5	2.2 (0.6–7.8)	0.176	9.3	**4.7 (2.2–11.1)**	**<0.001**	7.0	**2.1 (1.1–4.4)**	**0.024**	3.8	1.1 (0.4–2.4)	0.886	2.5	3.3 (0.8–15.4)	0.059
2006–2010	32.7	**3.7 (2.4–5.8)**	**<0.001**	4.4	**3.9 (1.2–13.7)**	**0.014**	3.3	2.9 (0.8–10.7)	0.080	10.4	**5.3 (2.3–12.8)**	**<0.001**	16.1	**4.9 (2.6–9.7)**	**<0.001**	5.2	1.4 (0.6–3.4)	0.375	0.5	0.7 (0.0–7.3)	0.844
2011–2015	38.5	**4.3 (2.8–6.8)**	**<0.001**	5.2	**4.6 (1.5–15.8)**	**0.004**	2.9	2.5 (0.6–9.7)	0.154	14.9	**7.6 (3.5–17.8)**	**<0.001**	14.9	**4.6 (2.4–9.1)**	**<0.001**	7.7	**2.2 (1.0–4.7)**	**0.044**	2.9	3.8 (0.8–19.3)	0.056
Age at study (years)																					
15–24	21.7	Ref.		2.8	Ref.		3.0	Ref.		7.9	Ref.		7.8	Ref.		5.9	Ref.		1.1	Ref.	
25–34	11.4	**0.5 (0.4–0.6)**	**<0.001**	1.2	**0.4 (0.2–0.7)**	**0.001**	1.7	**0.6 (0.3–1.0)**	**0.023**	3.3	**0.4 (0.3–0.6)**	**<0.001**	4.5	**0.6 (0.4–0.8)**	**<0.001**	3.1	**0.5 (0.4–0.8)**	**0.001**	0.7	0.6 (0.3–1.4)	0.247
35–44	10.4	**0.5 (0.4–0.6)**	**<0.001**	1.8	0.7 (0.3–1.2)	0.172	1.8	0.6 (0.3–1.1)	0.090	2.1	**0.3 (0.1–0.5)**	**<0.001**	5.0	**0.6 (0.4–0.9)**	**0.020**	2.4	**0.4 (0.2–0.7)**	**<0.001**	0.9	0.8 (0.3–2.1)	0.629
45–54	12.5	**0.6 (0.3–0.9)**	**0.011**	3.6	1.3 (0.5–2.7)	0.503	1.8	0.6 (0.2–1.6)	0.331	2.4	**0.3 (0.1–0.7)**	**0.002**	3.9	**0.5 (0.2–1.0)**	**0.036**	3.6	0.6 (0.2–1.3)	0.191	0.9	0.8 (0.1–3.4)	0.837
55–64	13.8	0.6 (0.0–3.6)	0.742	0.0	0.0 (0.0–12.2)	0.731	0.0	0.0 (0.0–11.5)	0.717	0.0	0.0 (0.0–4.3)	0.416	9.0	1.1 (0.0–6.5)	0.802	0.0	0.0 (0.0–8.8)	0.653	0.0	0.0 (0.0–33.7)	0.886

*Note*: Bold font indicates statistically significant difference as compared to the reference group at p < 0.05.

Abbreviations: CI, confidence interval; CNS, central nervous system; Ref., reference category; SCT, stem cell therapy.

**FIGURE 2:**
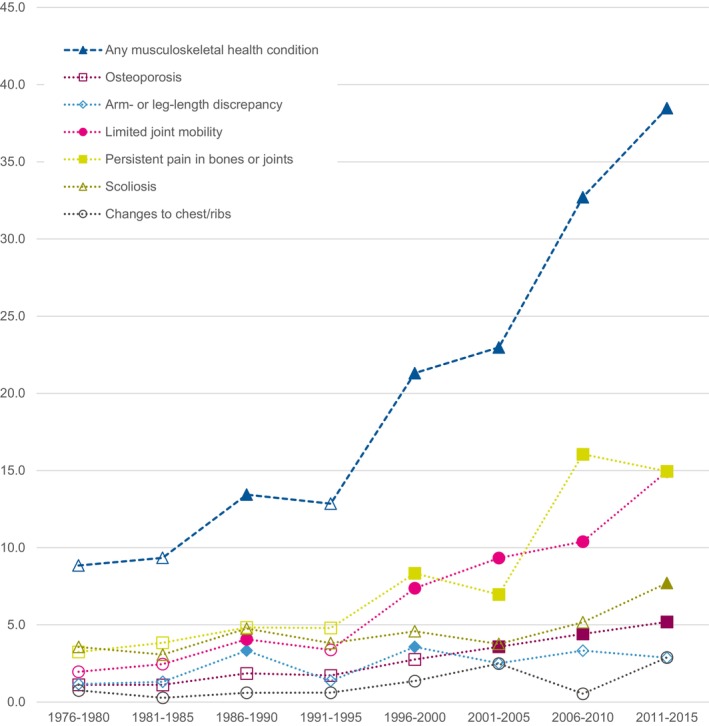
The incidence rates (per 1000 person‐years) of specific musculoskeletal health conditions stratified by year of diagnosis. Filled markers indicate statistically significantly increased rates as compared to the diagnostic period 1976–1980 at *p* < 0.05; empty markers indicate no statistically significant difference as compared to the diagnostic period 1976–1980.

We found that survivors diagnosed more recently had higher rates of *any MSHC* (diagnosed 2011–2015: RR = 4.3, 95%CI: 2.8–6.8) (Table 3; Figure 2). Older age at study was associated with lower rates of *any MSHC* (aged 35–44: RR = 0.5, 95%CI: 0.4–0.6; Table 3).

## 4 DISCUSSION

MSHC are prevalent in the survivors of childhood and adolescent cancers with increasing risk in younger cohorts and are commonly accompanied by other health conditions. Scoliosis, persistent pain in bones or joints and limited joint mobility are the most prevalent MSHC in this population. Risk for MSHCs is higher in female survivors, survivors of malignant bone tumours, survivors with a relapse, survivors who were aged 6–15 years at diagnosis, survivors treated with surgery, chemotherapy or stem cell therapy, and who were treated more recently.

Prevalence of *any MSHC* in survivors in our study (21%) was similar to previous studies. The North American Childhood Cancer Survivor Study (CCSS) found that 10.4% of survivors reported prevalence of *any MSHC*, but they only assessed four types of MSHCs.^12^ A single‐centre study from Switzerland found that 13.6% of CCS attending a follow‐up care visit had a MSHC,^18^ and a nationwide study from Poland found a prevalence of any MSHC of 19.2%.^17^ Regarding long‐term cumulative incidence of any MSHC, Bhakta and colleagues found that at the age of 50 years, 83.6% of survivors had experienced a MSHC,^13^ which is higher than what we observed in our cohort. This difference may be attributed to the younger average age of our cohort. However, comparison of overall MSHCs across studies is difficult because of differing treatment protocols, MSHC assessed, age distributions of the cohorts, and the number of specific MSHC that were assessed–with more MSHC being assessed and with older age of the cohort, the higher the overall prevalence of MSHC.

For the specific MSHCs, the prevalence for osteoporosis in our study (2.4%) was similar to the 2.6% reported in the CCSS.^12^ We found a higher proportion of survivors reporting prevalent arm‐ or leg‐length discrepancy (4.3%) than the CCSS (2.2%),^12^ but these differences may be caused by the assessment of outcomes: in the CCSS, survivors were asked whether they had ever had a ‘leg lengthening or shortening, or joint replacement’, which may be more restrictive than the question in our survey (‘arm‐ or leg‐length discrepancy’).

We are not aware of other studies that reported the prevalence of limited mobility of unspecified joints. However, a systematic review reported impaired ankle dorsiflexion in patients and survivors of childhood cancer.^28^ Further research is needed to clarify whether mobility limitations of other joints than the ankle joint are prevalent among CCS.

Various studies have investigated chronic pain in survivors,^29^, ^30^ but few with a specific focus on musculoskeletal pain: our study found that persistent pain in bones or joints was reported by 8% of survivors, which is lower than results from a study in Canada, which found that half of survivors who reported chronic pain (26.1%) located their pain in muscles or joints.^31^

Our finding that 8% of survivors report scoliosis supports the lower range of results from a systematic review (scoliosis: 10%–80%, kyphosis: 2%–48%).^2^ Prevalence of chest wall abnormalities (2%) was similar to that found in a recent systematic review (1.3%–2.2%).^32^ The prevalence of amputation or joint replacement in the CCSS was between 2.0%–2.7%,^11^ which is similar to our results (amputation: 2%, joint replacement: 1%).

Our study found an increased risk of osteoporosis and pain in bones and joints for female survivors. This relates to results from the general population that show musculoskeletal pain and osteoporosis being more prevalent in women.^33^, ^34^, ^35^ The IGHG guidelines for bone mineral density (BMD) surveillance found that osteoporosis risk is increased in male survivors.^15^ A cohort study of CCS from the Nordic countries (ALiCCS) found increased risk for osteoporosis in both male and female survivors, with a higher rate ratio in male survivors.^10^ One reason for the differences in findings could be that the ALiCCS study compared male survivors to male peers from the general population, and female survivors to female peers, whereas we directly compared male survivors to female survivors. The fact that osteoporosis is very rare in young men could then lead to higher rate ratios in men as compared to women. However, it is also possible that self‐report bias may play a role in our study, given the general health knowledge of osteoporosis occurring more often in women than men leading to more awareness for osteoporosis in women compared to men. This may have influenced self‐report of both male and female survivors.

After stratifying for diagnosis, we found that age at diagnosis remained a significant predictor of MSHC, with higher incidence rates in survivors aged 6–15 years at diagnosis as compared to those diagnosed at 0–5 years. More specifically, age at diagnosis seems to be a relevant factor in the development of subsequent osteoporosis, limited joint mobility and persistent pain in bones or joints in CCS. Regarding increased risk for joint mobility deficits and pain in bones or joints, studies show that osteonecrosis risk is increased in children older at childhood cancer treatment (>10 years),^3^ and is highest in leukaemia survivors.^10^ The leading symptom of osteonecrosis is pain in the affected joint, which may contribute to the increased risk for limited joint mobility and pain in bones and joints that we found in leukaemia survivors aged >10 years at diagnosis. However, as osteonecrosis is rare (prevalence of 2.5% in leukaemia survivors),^36^ this can explain only a small fraction of the symptoms in our cohort. Other reasons for the impact of age at diagnosis on development of MSHC could be that the musculoskeletal system may be more vulnerable during the pubertal growth spurt, or that the bodies of younger children may have a higher biological resilience to injuries of the musculoskeletal system.

Another study has also investigated temporal trends in the prevalence of MSHC: Mulrooney and colleagues found that low BMD and osteonecrosis were becoming more frequent in survivors of acute lymphoblastic leukaemia with current treatment protocols as compared to those treated according to older protocols.^19^ Our study provides evidence from an unselected cohort of CCS and found increasing incidence rates of MSHC in more recently diagnosed and treated survivors. This was most pronounced for osteoporosis, limited joint mobility, and persistent pain in bones or joints. Our results are in accordance with the results of Mulrooney et al., as increased frequency of osteonecrosis might partly explain the increased rates of bone and joint pain, as well as limited joint mobility. Also, with new treatment modalities, survival rates are increasing and life‐threatening late effects are decreasing,^19^, ^37^ but this may in turn result in more survivors having poorer health.^37^ In accordance with this, a recent study of the CCSS showed increased rates of late major surgery of survivors diagnosed in the 1990s as compared to those diagnosed in the 1970s.^38^ Another possibility is that survivors are now better informed and more aware of potential late effects and may be more likely to report them, or that recall bias may play a role for those diagnosed less recently. Temporal trends in MSHC may therefore be partly real and partly artefactual and we would encourage longitudinal studies that investigate MSHCs from diagnosis to long‐term follow‐up care using objective measures.

Given that MSHC are prevalent in CCS, with increasing risk in cohorts diagnosed more recently, and usually occurring in multimorbid survivors, there is a need to strengthen rehabilitation services for survivors to address these challenges. MSHC are associated with impaired physical functioning,^12^ and lower quality of life in survivors.^9^ The goal of rehabilitation is to improve physical functioning,^39^ as well as prevention of secondary complications that can occur in persons with MSHC, for example, back pain, low‐trauma fractures, or gait abnormalities.^14^, ^15^ Several studies have highlighted the importance of rehabilitation in mitigating the impact of the cancer treatment and improving physical functioning of survivors.^16^, ^40^, ^41^ Even though guidelines recommend an assessment of children and adolescents with cancer by a rehabilitation professional,^42^ evidence suggests that rehabilitation services are currently underutilised in this population.^43^, ^44^, ^45^, ^46^ Therefore, it is important to assess MSHCs in survivors during follow‐up care, as follow‐up care providers can initiate referral to rehabilitation services if indicated.

One strength of our study is its nationwide, population‐based design: Rueegg et al. showed that results of the SCCSS have low risk of nonresponse bias and are generalizable to the population of CCS in Switzerland.^47^ Other strengths are the good response rate of the SCCSS, the availability of objective diagnostic and treatment‐related information from the ChCR, and the detailed assessment of specific MSHC. A limitation of this study are the self‐reported primary outcomes, which introduce the potential for information bias. However, our results are similar to other studies in the field, which supports the validity of our results. Another limitation is the lack of a control group. However, results from the SCCSS have shown that prevalence of musculoskeletal and neurological health conditions (combined) in survivors was statistically significantly higher than in comparisons^9^—a finding that is consistent with the CCSS.^11^

In conclusion, MSHC are prevalent in survivors of childhood and adolescent cancers, risk is higher for more recent survivor cohorts, and MSHC usually occur in multimorbid survivors. Strengthening rehabilitation services, awareness of follow‐up care providers, and appropriate referral to rehabilitation specialists are needed to mitigate the effects of the cancer and its treatment and improve survivors' physical functioning and quality of life.

## AUTHOR CONTRIBUTIONS

**Salome Christen:** Conceptualization (equal); formal analysis (equal); funding acquisition (equal); methodology (equal); project administration (equal); software (equal); validation (equal); visualization (lead); writing – original draft (lead); writing – review and editing (lead). **Katharina Roser:** Conceptualization (equal); methodology (equal); supervision (equal); validation (equal); writing – review and editing (equal). **Luzius Mader:** Data curation (equal); investigation (equal); supervision (equal); validation (equal); writing – review and editing (equal). **Maria Otth:** Resources (equal); validation (equal); writing – review and editing (equal). **Katrin Scheinemann:** Resources (equal); validation (equal); writing – review and editing (equal). **Grit Sommer:** Data curation (equal); investigation (equal); validation (equal); writing – review and editing (equal). **Claudia Kuehni:** Conceptualization (equal); data curation (equal); funding acquisition (equal); investigation (equal); methodology (equal); resources (equal); validation (equal); writing – review and editing (equal). **Gisela Michel:** Conceptualization (equal); funding acquisition (equal); investigation (equal); methodology (equal); resources (equal); supervision (equal); validation (equal); writing – review and editing (equal).

## FUNDING INFORMATION

This study was financially supported by the Swiss Cancer League and Swiss Cancer Research (KLS/KFS‐4825‐01‐2019, KFS‐4722‐02‐2019, HSR‐4951‐11‐2019, KFS‐5027‐02‐2020, KLS/KFS‐5711‐01‐2022), Kinderkrebshilfe Schweiz (www.kinderkrebshilfe.ch), and Stiftung für krebskranke Kinder—Regio basiliensis. SC has received funding from Vontobel‐Stiftung, Faculty of Health Sciences and Medicine of the University of Lucerne, Krebsliga Zentralschweiz, Avenira Stiftung and one anonymous foundation.

## CONFLICT OF INTEREST STATEMENT

The authors declare no conflicts of interest.

## ETHICS STATEMENT

Ethical approval of the SCCSS was granted by the Ethics Committee of the Canton of Bern (KEK‐No. 166/14 and 2021–01462).

## Supporting information


Data S1:

## Data Availability

The data that support the information of this manuscript were accessed on secured servers of the Institute of Social and Preventive Medicine at the University of Bern. Individual‐level, fully anonymized, sensitive data can only be made available for researchers who fulfil the respective legal requirements. Requests of data from the Childhood Cancer Registry must be directed to the Childhood Cancer Registry of Switzerland (https://www.childhoodcancerregistry.ch/). Requests of data from the Swiss Childhood Cancer Survivor Study (SCCSS) should be communicated to the study lead Claudia E. Kuehni (claudia.kuehni@unibe.ch).

## References

[1] Ji T , Hayden JB , Hoang BH . Musculoskeletal system. Specialty Topics in Pediatric Neuropsychology, Handbook of Long Term Care of the Childhood Cancer Survivor. 1st ed. Springer US; 2015:155‐170.

[2] Gawade PL , Hudson MM , Kaste SC , et al. A systematic review of selected musculoskeletal late effects in survivors of childhood cancer. Curr Pediatr Rev. 2014;10(4):249‐262. doi:10.2174/1573400510666141114223827 25403639 PMC4336580

[3] Marcus R Jr , Esiashvili N . Musculoskeletal, integument. Pediatric Oncology, Survivors of Childhood and Adolescent Cancer. A Multidisciplinary Approach. 3rd ed. Springer International Publishing; 2015:297‐324.

[4] Goodenough CG , Partin RE , Ness KK . Skeletal muscle and childhood cancer: where are we now and where we go from here. Aging Cancer. 2021;2(1–2):13‐35. doi:10.1002/aac2.12027 34541550 PMC8445321

[5] Krivitzky LS , Blaufuss MM , VanDenHeuvel S . Rehabilitation consideration in pediatric cancer survivors. Specialty Topics in Pediatric Neuropsychology, Handbook of Long Term Care of the Childhood Cancer Survivor. 1st ed. Springer US; 2015:385‐395.

[6] Erdmann F , Frederiksen LE , Bonaventure A , et al. Childhood cancer: survival, treatment modalities, late effects and improvements over time. Cancer Epidemiol. 2020;71:101733. doi:10.1016/j.canep.2020.101733 32461035

[7] Vos T , Abajobir AA , Abate KH , et al. Global, regional, and national incidence, prevalence, and years lived with disability for 328 diseases and injuries for 195 countries, 1990–2016: a systematic analysis for the global burden of disease study 2016. Lancet. 2017;390(10100):1211‐1259. doi:10.1016/S0140-6736(17)32154-2 28919117 PMC5605509

[8] Cieza A , Causey K , Kamenov K , Hanson SW , Chatterji S , Vos T . Global estimates of the need for rehabilitation based on the global burden of disease study 2019: a systematic analysis for the global burden of disease study 2019. Lancet. 2020;396(10267):2006‐2017. doi:10.1016/S0140-6736(20)32340-0 33275908 PMC7811204

[9] Rueegg CS , Gianinazzi ME , Rischewski J , et al. Health‐related quality of life in survivors of childhood cancer: the role of chronic health problems. J Cancer Surviv. 2013;7(4):511‐522. doi:10.1007/s11764-013-0288-4 23784593

[10] Oskarsson T , Duun‐Henriksen AK , Bautz A , et al. Skeletal adverse events in childhood cancer survivors: an adult life after childhood cancer in Scandinavia (ALiCCS) cohort study. Int J Cancer. 2021;149:1863‐1876. doi:10.1002/ijc.33741 34278568

[11] Suh E , Stratton KL , Leisenring WM , et al. Late mortality and chronic health conditions in long‐term survivors of early‐adolescent and young adult cancers: a retrospective cohort analysis from the childhood cancer survivor study. Lancet Oncol. 2020;21(3):421‐435. doi:10.1016/s1470-2045(19)30800-9 32066543 PMC7392388

[12] Ness KK , Mertens AC , Hudson MM , et al. Limitations on physical performance and daily activities among long‐term survivors of childhood cancer. Ann Intern Med. 2005;143(9):639‐647. doi:10.7326/0003-4819-143-9-200511010-00007 16263886

[13] Bhakta N , Liu Q , Ness KK , et al. The cumulative burden of surviving childhood cancer: an initial report from the St Jude lifetime cohort study (SJLIFE). Lancet. 2017;390(10112):2569‐2582. doi:10.1016/S0140-6736(17)31610-0 28890157 PMC5798235

[14] Survivors of Childhood and Adolescent Cancer . In: Schwartz CL , Hobbie WL , Constine LS , Ruccione KS , eds. A Multidisciplinary Approach. 3rd ed. Springer International Publishing; 2015.

[15] van Atteveld JE , Mulder RL , van den Heuvel‐Eibrink MM , et al. Bone mineral density surveillance for childhood, adolescent, and young adult cancer survivors: evidence‐based recommendations from the international late effects of childhood cancer guideline harmonization group. Lancet Diabetes Endocrinol. 2021;9:622‐637. doi:10.1016/S2213-8587(21)00173-X 34339631 PMC8744935

[16] Tanner L , Keppner K , Lesmeister D , Lyons K , Rock K , Sparrow J . Cancer rehabilitation in the pediatric and adolescent/young adult population. Semin Oncol Nurs. 2020;36(1):150984. doi:10.1016/j.soncn.2019.150984 31983485

[17] Latoch E , Zubowska M , Młynarski W , et al. Late effects of childhood cancer treatment in long‐term survivors diagnosed before the age of 3 years–a multicenter, nationwide study. Cancer Epidemiol. 2022;80:102209. doi:10.1016/j.canep.2022.102209 35868173

[18] Babecoff S , Mermillod F , Marino D , et al. Long‐term follow‐up for childhood cancer survivors: the Geneva experience. Swiss Med Wkly. 2022;152:w30153. doi:10.4414/smw.2022.w30153 35429234

[19] Mulrooney DA , Hyun G , Ness KK , et al. The changing burden of long‐term health outcomes in survivors of childhood acute lymphoblastic leukaemia: a retrospective analysis of the St Jude Lifetime Cohort Study. Lancet Haematol. 2019;6(6):e306‐e316. doi:10.1016/S2352-3026(19)30050-X 31078468 PMC6756152

[20] van Dijk IWEM , Oldenburger F , Cardous‐Ubbink MC , et al. Evaluation of late adverse events in long‐term wilms' tumor survivors. Int J Radiat Oncol Biol Phys. 2010;78(2):370‐378. doi:10.1016/j.ijrobp.2009.08.016 20137867

[21] Landier W , Skinner R , Wallace WH , et al. Surveillance for late effects in childhood cancer survivors. J Clin Oncol. 2018;36(21):2216‐2222. doi:10.1200/JCO.2017.77.0180 29874139 PMC6804892

[22] Marina NM , Liu Q , Donaldson SS , et al. Longitudinal follow‐up of adult survivors of Ewing sarcoma: a report from the childhood cancer survivor study. Cancer. 2017;123(13):2551‐2560. doi:10.1002/cncr.30627 28222219 PMC5474122

[23] Laverdière C , Liu Q , Yasui Y , et al. Long‐term outcomes in survivors of neuroblastoma: a report from the childhood cancer survivor study. J Natl Cancer Inst. 2009;101(16):1131‐1140. doi:10.1093/jnci/djp230 19648511 PMC2728747

[24] Michel G , von der Weid N , Zwahlen M , et al. The Swiss childhood cancer registry: rationale, organisation and results for the years 2001‐2005. Swiss Med Wkly. 2007;137(35–36):502‐509. 17990137 10.4414/smw.2007.11875

[25] Kuehni CE , Rueegg CS , Michel G , et al. Cohort profile: the Swiss childhood cancer survivor study. Int J Epidemiol. 2012;41(6):1553‐1564. doi:10.1093/ije/dyr142 22736394

[26] Steliarova‐Foucher E , Stiller C , Lacour B , Kaatsch P . International classification of childhood cancer, third edition. Cancer. 2005;103(7):1457‐1467. doi:10.1002/cncr.20910 15712273

[27] StataCorp . Stata Statistical Software: Release 18. StataCorp LLC; 2023.

[28] Beulertz J , Wurz A , Culos‐Reed N , Chamorro Viña C , Bloch W , Baumann FT . Ankle dorsiflexion in childhood cancer patients: a review of the literature. Cancer Nurs. 2015;38(6):447‐457. doi:10.1097/NCC.0000000000000223 25730588

[29] Reinfjell T , Zeltzer L . A systematic review of self‐reported pain in childhood cancer survivors. Acta Paediatrica (Oslo, Norway: 1992). 2020;109(1):56‐70. doi:10.1111/apa.14977 31423647

[30] Schulte FSM , Patton M , Alberts NM , et al. Pain in long‐term survivors of childhood cancer: a systematic review of the current state of knowledge and a call to action from the Children's Oncology Group. Cancer. 2021;127(1):35‐44. doi:10.1002/cncr.33289 33112416 PMC7875461

[31] Patton M , Forster VJ , Forbes C , et al. Characterizing pain in long‐term survivors of childhood cancer. Support Care Cancer. 2021;30:295‐303. doi:10.1007/s00520-021-06386-4 34278531

[32] Kasteler R , Lichtensteiger C , Schindera C , Ansari M , Kuehni CE . Validation of questionnaire‐reported chest wall abnormalities with a telephone interview in Swiss childhood cancer survivors. BMC Cancer. 2021;21(1):787. doi:10.1186/s12885-021-08425-z 34238236 PMC8268220

[33] Kanis JA , McCloskey EV , Johansson H , Oden A , Melton LJ , Khaltaev N . A reference standard for the description of osteoporosis. Bone. 2008;42(3):467‐475. doi:10.1016/j.bone.2007.11.001 18180210

[34] Cimas M , Ayala A , Sanz B , Agulló‐Tomás MS , Escobar A , Forjaz MJ . Chronic musculoskeletal pain in European older adults: cross‐national and gender differences. Eur J Pain. 2018;22(2):333‐345. doi:10.1002/ejp.1123 29235193

[35] Salari N , Ghasemi H , Mohammadi L , et al. The global prevalence of osteoporosis in the world: a comprehensive systematic review and meta‐analysis. J Orthop Surg Res. 2021;16(1):609. doi:10.1186/s13018-021-02772-0 34657598 PMC8522202

[36] Girard P , Auquier P , Barlogis V , et al. Symptomatic osteonecrosis in childhood leukemia survivors: prevalence, risk factors and impact on quality of life in adulthood. Haematologica. 2013;98(7):1089‐1097. doi:10.3324/haematol.2012.081265 23645686 PMC3696613

[37] Ness KK , Hudson MM , Jones KE , et al. Effect of temporal changes in therapeutic exposure on self‐reported health status in childhood cancer survivors. Ann Intern Med. 2017;166(2):89‐98. doi:10.7326/M16-0742 27820947 PMC5239750

[38] Dieffenbach BV , Murphy AJ , Liu Q , et al. Cumulative burden of late, major surgical intervention in survivors of childhood cancer: a report from the childhood cancer survivor study (CCSS) cohort. Lancet Oncol. 2023;24(6):691‐700. doi:10.1016/S1470-2045(23)00154-7 37182536 PMC10348667

[39] Negrini S , Selb M , Kiekens C , et al. Rehabilitation definition for research purposes. A global stakeholders' initiative by cochrane rehabilitation. Neurorehabil Neural Repair. 2022;36:405‐414. doi:10.1177/15459683221093587 35574944

[40] Pruitt DW , Haas MT , Bolikal PD . Functional impairment in pediatric cancer survivorship. Pediatr Clin N Am. 2023;70(3):501‐515. doi:10.1016/j.pcl.2023.01.002 37121639

[41] Riedl D , Licht T , Nickels A , et al. Large improvements in health‐related quality of life and physical fitness during multidisciplinary inpatient rehabilitation for pediatric cancer survivors. Cancers (Basel). 2022;14(19):4855. doi:10.3390/cancers14194855 36230777 PMC9563065

[42] L'Hotta AJ , Randolph SB , Reader B , Lipsey K , King AA . Clinical practice guideline and expert consensus recommendations for rehabilitation among children with cancer: a systematic review. CA Cancer J Clin. 2023;73:524‐545. doi:10.3322/caac.21783 37158423 PMC10524286

[43] Gohar SF , Marchese V , Comito M . Physician referral frequency for physical therapy in children with acute lymphoblastic leukemia. Pediatr Hematol Oncol. 2010;27(3):179‐187. doi:10.3109/08880010903580209 20367261

[44] Ospina PA , Wiart L , Eisenstat DD , McNeely ML . Physical rehabilitation practices for children and adolescents with cancer in Canada. Physiother Can. 2020;72(2):207‐216. doi:10.3138/ptc-2018-0077 32494104 PMC7238940

[45] Rodwin RL , Ma X , Ness KK , Kadan‐Lottick NS , Wang R . Physical therapy utilization among hospitalized patients with pediatric acute lymphoblastic leukemia. JCO Oncol Pract. 2022;18:e1060‐e1068. doi:10.1200/OP.21.00796 35427182 PMC9287366

[46] Montgomery M , Huang S , Cox CL , et al. Physical therapy and chiropractic use among childhood cancer survivors with chronic disease: impact on health‐related quality of life. J Cancer Surviv. 2011;5(1):73‐81. doi:10.1007/s11764-010-0151-9 20922492 PMC3062253

[47] Rueegg CS , Gianinazzi ME , Michel G , Zwahlen M , von der Weid NX , Kuehni CE . No evidence of response bias in a population‐based childhood cancer survivor questionnaire survey–results from the Swiss childhood cancer survivor study. PLoS One. 2017;12(5):e0176442. doi:10.1371/journal.pone.0176442 28463966 PMC5413049

