# Supplementary material for: Incidence and prevalence of musculoskeletal health conditions in survivors of childhood and adolescent cancers: A report from the Swiss childhood cancer survivor study
Source: Cancer Med. 2024 Apr 23;13(8):e7204. doi: 10.1002/cam4.7204 (PMC11036073; doi:10.1002/cam4.7204)
Supplement: Supplementary file 1 — Data S1: [file CAM4-13-e7204-s001.docx]

Incidence and prevalence of musculoskeletal health conditions in survivors of childhood and adolescent cancers: A report from the Swiss Childhood Cancer Survivor Study.

Salome Christen^1^, Katharina Roser^1^, Luzius Mader^2^, Maria Otth^1,3,4^, Katrin Scheinemann^1,4,5^, Grit Sommer^6^, Claudia Kuehni^6,7^, Gisela Michel^1^

^1^Faculty of Health Sciences and Medicine, University of Lucerne, Lucerne, Switzerland

^2^Cancer Registry Bern Solothurn, University of Berne, Berne, Switzerland

^3^Department of Oncology, University Children's Hospital Zurich, Zurich, Switzerland

^4^Division of Pediatric Hematology/Oncology, Children's Hospital of Eastern Switzerland, St. Gallen, Switzerland

^5^Department of Pediatrics, McMaster Children's Hospital and McMaster University, Hamilton, ON, Canada

^6^Swiss Childhood Cancer Registry, Institute of Social and Preventive Medicine, University of Bern, Switzerland

^7^Division of Pediatric Hematology/Oncology, Department of Pediatrics, Inselspital, Bern University Hospital, University of Bern, Bern, Switzerland

**Corresponding author:** Salome Christen Faculty of Health Sciences and Medicine, University of Lucerne, Lucerne, Switzerland. E-Mail: [salome.christen@unilu.ch](mailto:salome.christen@unilu.ch)

Supplemental Table 1. Number of missing values in outcomes of Table 2.

|  | Prevalence  N= (%) | Cumulative Incidence  N= (%) | Condition no longer prevalent^a^  N= (%) | Age at incidence^b^  N= (%) | Time since diagnosis at incidence^c^  N= (%) |
| --- | --- | --- | --- | --- | --- |
| Any musculoskeletal health condition | 129 (4.9) | 190 (7.2) | n.a. | 125 (19.4) | 125 (19.4) |
| Osteoporosis | 129 (4.9) | 130 (4.9) | n.a. | 19 (18.4) | 19 (18.4) |
| Arm- or leg-length discrepancy | 129 (4.9) | 142 (5.4) | n.a. | 33 (27.3) | 33 (27.3) |
| Limited joint mobility | 129 (4.9) | 135 (5.1) | n.a. | 36 (16.0) | 36 (16.0) |
| Persistent pain in bones or joints | 129 (4.9) | 132 (5.0) | n.a. | 50 (17.9) | 50 (17.9) |
| Scoliosis | 129 (4.9) | 166 (6.3) | n.a. | 73 (31.7) | 73 (31.7) |
| Changes to chest/ribs | 129 (4.9) | 135 (5.1) | n.a. | 14 (28.0) | 14 (28.0) |
| Any musculoskeletal surgery | n.a. | 202 (7.6) | n.a. | n.a. | n.a. |
| Amputation | n.a. | 205 (7.8) | n.a. | n.a. | n.a. |
| Rotationplasty | n.a. | 204 (7.7) | n.a. | n.a. | n.a. |
| Joint replacement or arthrodesis | n.a. | 204 (7.7) | n.a. | n.a. | n.a. |
| Limb lengthening/shortening | n.a. | 205 (7.8) | n.a. | n.a. | n.a. |
| Scoliosis surgery or spondylodesis | n.a. | 205 (7.8) | n.a. | n.a. | n.a. |
| Thorax surgery | n.a. | 205 (7.8) | n.a. | n.a. | n.a. |
| Fracture surgery | n.a. | 205 (7.8) | n.a. | n.a. | n.a. |
| Any other MSK surgery | n.a. | 204 (7.7) | n.a. | n.a. | n.a. |

Abbreviations: MSK=musculoskeletal, n.a.=not applicable

^a^ Number of survivors that indicated cumulative incidence 'yes' and prevalence 'no'. See missings listed under 'Prevalence' and 'Cumulative Incidence' separately.

^b^ Missing values in 'age at incidence' are only displayed for survivors who had cumulative incidence: yes (see **Table 2**)

^c^ Missing values in 'time since diagnosis at incidence' are only displayed for survivors who had cumulative incidence: yes (see **Table 2**)

Supplemental Table 2. Cumulative incidence and risk ratios of any and the specific musculoskeletal health conditions in adolescent and adult survivors of childhood and adolescent cancer (unadjusted; total sample size: N=2645).

|  | | **Any musculoskeletal health condition** | | | **Osteoporosis** | | | **Arm- or leg-length discrepancy** | | | **Limited joint mobility** | | | **Persistent pain in bones or joints** | | | **Scoliosis** | | | **Changes to chest/ribs** | | |
| --- | --- | --- | --- | --- | --- | --- | --- | --- | --- | --- | --- | --- | --- | --- | --- | --- | --- | --- | --- | --- | --- | --- |
|  |  | Cumulative incidence | Risk ratios  (95% CI) | p-value | Cumulative incidence | Risk ratios  (95% CI) | p-value | Cumulative incidence | Risk ratios  (95% CI) | p-value | Cumulative incidence | Risk ratios  (95% CI) | p-value | Cumulative incidence | Risk ratios  (95% CI) | p-value | Cumulative incidence | Risk ratios  (95% CI) | p-value | Cumulative incidence | Risk ratios  (95% CI) | p-value |
|  | Overall | 26.2 | n.a. |  | 4.1 | n.a. |  | 4.8 | n.a. |  | 9.0 | n.a. |  | 11.1 | n.a. |  | 9.3 | n.a. |  | 2.0 | n.a. |  |
| **Sex** | Males | 22.8 | Ref. |  | 2.8 | Ref. |  | 4.0 | Ref. |  | 8.2 | Ref. |  | 9.1 | Ref. |  | 8.0 | Ref. |  | 2.3 | Ref. |  |
|  | Females | 29.8 | **1.3 (1.14-1.49)** | **<0.001** | 5.5 | **1.9 (1.31-2.89)** | **0.001** | 5.8 | **1.5 (1.02-2.06)** | **0.037** | 9.8 | 1.2 (0.92-1.52) | 0.187 | 13.3 | **1.5 (1.16-1.82)** | **0.001** | 10.6 | **1.3 (1.03-1.69)** | **0.028** | 1.7 | 0.7 (0.42-1.28) | 0.267 |
| **Diagnosis** | Leukaemia | 25.4 | Ref. |  | 5.4 | Ref. |  | 2.6 | Ref. |  | 7.9 | Ref. |  | 12.5 | Ref. |  | 6.4 | Ref. |  | 0.8 | Ref. |  |
|  | Lymphoma | 21.6 | 0.9 (0.69-1.05) | 0.124 | 2.5 | **0.5 (0.26-0.86)** | **0.011** | 1.8 | 0.7 (0.32-1.47) | 0.328 | 4.9 | 0.6 (0.40-0.97) | 0.033 | 8.9 | **0.7 (0.51-0.99)** | **0.042** | 6.5 | 1.0 (0.66-1.54) | 0.985 | 3.3 | **3.9 (1.58-9.86)** | **0.002** |
|  | CNS tumour | 25.3 | 1.0 (0.80-1.23) | 0.965 | 5.5 | 1.0 (0.62-1.70) | 0.930 | 4.3 | 1.6 (0.86-3.12) | 0.128 | 9.1 | 1.1 (0.77-1.71) | 0.497 | 8.6 | **0.7 (0.47-1.00)** | **0.045** | 11.7 | **1.8 (1.22-2.68)** | **0.003** | 2.3 | **2.7 (0.98-7.64)** | **0.045** |
|  | Neuroblastoma | 28.4 | 1.1 (0.77-1.62) | 0.557 | 2.4 | 0.5 (0.11-1.83) | 0.246 | 7.4 | **2.8 (1.15-6.82)** | **0.019** | 7.3 | 0.9 (0.41-2.08) | 0.848 | 9.8 | 0.8 (0.39-1.55) | 0.472 | 22.0 | **3.4 (2.08-5.59)** | **<0.001** | 1.2 | 1.5 (0.18-12.01) | 0.722 |
|  | Retinoblastoma | 19.5 | 0.8 (0.41-1.45) | 0.399 | 0.0 | 0.0 (.-.) | 0.126 | 4.9 | 1.8 (0.45-7.66) | 0.396 | 7.3 | 0.9 (0.30-2.83) | 0.890 | 12.2 | 1.0 (0.42-2.27) | 0.954 | 9.8 | 1.5 (0.57-4.00) | 0.407 | 0.0 | 0.0 (.-.) | 0.557 |
|  | Renal tumour | 27.0 | 1.1 (0.76-1.48) | 0.712 | 1.7 | 0.3 (0.08-1.31) | 0.090 | 7.1 | **2.7 (1.20-5.97)** | **0.013** | 5.2 | 0.7 (0.29-1.49) | 0.309 | 7.0 | 0.6 (0.28-1.13) | 0.091 | 15.8 | **2.5 (1.47-4.07)** | **0.001** | 2.6 | 3.1 (0.79-12.34) | 0.087 |
|  | Hepatic tumour | 14.3 | 0.6 (0.16-2.04) | 0.343 | 0.0 | 0.0 (.-.) | 0.354 | 0.0 | 0.0 (.-.) | 0.524 | 6.7 | 0.8 (0.12-5.69) | 0.859 | 6.7 | 0.5 (0.08-3.58) | 0.497 | 0.0 | 0.0 (.-.) | 0.326 | 0.0 | 0.0 (.-.) | 0.723 |
|  | Malignant bone tumour | 64.4 | **2.5 (2.12-3.03)** | **<0.001** | 3.7 | 0.7 (0.27-1.69) | 0.399 | 31.1 | **11.8 (7.07-19.60)** | **<0.001** | 45.6 | **5.8 (4.23-7.85)** | **<0.001** | 30.1 | **2.4 (1.75-3.32)** | **<0.001** | 16.5 | **2.6 (1.60-4.12)** | **<0.001** | 3.7 | **4.4 (1.37-14.25)** | **0.007** |
|  | Soft tissue sarcoma | 28.2 | 1.1 (0.84-1.47) | 0.466 | 1.9 | 0.3 (0.11-1.11) | 0.057 | 6.3 | **2.4 (1.13-5.02)** | **0.020** | 10.8 | 1.4 (0.81-2.27) | 0.244 | 8.2 | 0.7 (0.38-1.14) | 0.125 | 11.9 | **1.9 (1.12-3.08)** | **0.017** | 2.5 | 3.0 (0.86-10.51) | 0.072 |
|  | Germ cell tumour | 16.5 | **0.7 (0.44-0.97)** | **0.025** | 4.9 | 0.9 (0.42-1.99) | 0.813 | 1.4 | 0.5 (0.13-2.26) | 0.384 | 2.1 | **0.3 (0.09-0.85)** | **0.014** | 7.7 | 0.6 (0.34-1.13) | 0.107 | 4.3 | 0.7 (0.29-1.53) | 0.329 | 1.4 | 1.7 (0.34-8.29) | 0.514 |
|  | Other tumour | 21.6 | 0.9 (0.54-1.34) | 0.477 | 6.6 | 1.2 (0.49-2.99) | 0.673 | 3.9 | 1.5 (0.45-4.93) | 0.510 | 2.6 | 0.3 (0.08-1.33) | 0.094 | 7.9 | 0.6 (0.29-1.39) | 0.241 | 9.5 | 1.5 (0.69-3.13) | 0.324 | 1.3 | 1.6 (0.19-12.94) | 0.668 |
|  | Langerhans cell histiocytosis | 24.4 | 1.0 (0.64-1.45) | 0.843 | 5.0 | 0.9 (0.34-2.52) | 0.875 | 2.5 | 1.0 (0.23-4.04) | 0.953 | 6.3 | 0.8 (0.33-1.91) | 0.597 | 17.3 | 1.4 (0.83-2.31) | 0.225 | 12.3 | **1.9 (1.01-3.65)** | **0.049** | 1.2 | 1.5 (0.18-12.15) | 0.713 |
| **Relapse** | No | 24.4 | Ref. |  | 3.6 | Ref. |  | 4.8 | Ref. |  | 7.8 | Ref. |  | 10.3 | Ref. |  | 8.5 | Ref. |  | 1.8 | Ref. |  |
|  | Yes | 32.9 | **1.3 (1.16-1.56)** | **<0.001** | 6.0 | **1.7 (1.10-2.49)** | **0.016** | 4.8 | 1.0 (0.65-1.53) | 0.992 | 13.5 | **1.7 (1.33-2.26)** | **<0.001** | 14.3 | **1.4 (1.08-1.77)** | **0.011** | 12.3 | **1.4 (1.10-1.89)** | **0.009** | 2.7 | 1.5 (0.81-2.74) | 0.199 |
| **Age at diagnosis** | 0-5 years at dx | 25.2 | Ref. |  | 3.1 | Ref. |  | 5.2 | Ref. |  | 6.6 | Ref. |  | 9.7 | Ref. |  | 12.4 | Ref. |  | 2.1 | Ref. |  |
|  | 6-10 years at dx | 28.2 | 1.1 (0.93-1.34) | 0.225 | 3.7 | 1.2 (0.68-2.16) | 0.524 | 6.9 | 1.3 (0.86-2.02) | 0.209 | 9.6 | **1.5 (1.01-2.10)** | **0.044** | 9.8 | 1.0 (0.73-1.41) | 0.925 | 10.2 | 0.8 (0.61-1.13) | 0.225 | 1.1 | 0.5 (0.20-1.26) | 0.132 |
|  | 11-15 years at dx | 27.4 | 1.1 (0.92-1.29) | 0.338 | 5.1 | **1.7 (1.02-2.79)** | **0.039** | 3.9 | 0.7 (0.47-1.18) | 0.210 | 12.1 | **1.8 (1.32-2.54)** | **<0.001** | 13.1 | **1.4 (1.02-1.80)** | **0.033** | 7.6 | **0.6 (0.45-0.84)** | **0.002** | 2.2 | 1.0 (0.53-2.03) | 0.923 |
|  | 16-20 years at dx | 22.9 | 0.9 (0.73-1.13) | 0.390 | 4.6 | 1.5 (0.83-2.73) | 0.177 | 3.1 | 0.6 (0.33-1.12) | 0.103 | 6.3 | 1.0 (0.60-1.51) | 0.840 | 11.9 | 1.2 (0.87-1.73) | 0.235 | 5.3 | **0.4 (0.27-0.67)** | **<0.001** | 2.7 | 1.3 (0.59-2.69) | 0.548 |
| **Surgery** | No | 24.9 | Ref. |  | 5.5 | Ref. |  | 2.4 | Ref. |  | 7.1 | Ref. |  | 12.1 | Ref. |  | 6.7 | Ref. |  | 0.9 | Ref. |  |
|  | Yes | 27.2 | 1.1 (0.94-1.26) | 0.252 | 3.7 | **0.7 (0.45-0.97)** | **0.036** | 5.9 | **2.5 (1.53-4.11)** | **<0.001** | 10.0 | **1.4 (1.05-1.89)** | **0.021** | 10.7 | 0.9 (0.70-1.12) | 0.316 | 10.5 | **1.6 (1.17-2.14)** | **0.003** | 2.4 | **2.6 (1.15-5.68)** | **0.017** |
| **Chemotherapy** | No | 22.1 | Ref. |  | 3.5 | Ref. |  | 4.1 | Ref. |  | 6.6 | Ref. |  | 8.7 | Ref. |  | 10.9 | Ref. |  | 1.2 | Ref. |  |
|  | Yes | 27.8 | **1.3 (1.05-1.51)** | **0.010** | 4.4 | 1.3 (0.77-2.09) | 0.348 | 5.1 | 1.2 (0.78-1.96) | 0.367 | 9.9 | **1.5 (1.05-2.13)** | **0.022** | 11.9 | **1.4 (1.00-1.85)** | **0.045** | 8.9 | 0.8 (0.61-1.09) | 0.168 | 2.2 | 1.9 (0.79-4.35) | 0.146 |
| **Radiotherapy** | No | 26.2 | Ref. |  | 4.2 | Ref. |  | 4.6 | Ref. |  | 9.4 | Ref. |  | 11.5 | Ref. |  | 8.1 | Ref. |  | 1.5 | Ref. |  |
|  | Yes | 27.2 | 1.0 (0.90-1.20) | 0.594 | 4.5 | 1.1 (0.72-1.59) | 0.748 | 5.1 | 1.1 (0.77-1.62) | 0.578 | 8.4 | 0.9 (0.68-1.18) | 0.435 | 10.4 | 0.9 (0.71-1.15) | 0.418 | 11.9 | **1.5 (1.14-1.90)** | **0.003** | 2.7 | **1.8 (1.02-3.19)** | **0.042** |
| **SCT** | No | 26.4 | Ref. |  | 4.2 | Ref. |  | 4.9 | Ref. |  | 9.0 | Ref. |  | 11.1 | Ref. |  | 9.3 | Ref. |  | 1.8 | Ref. |  |
|  | Yes | 33.3 | 1.3 (0.93-1.72) | 0.156 | 6.9 | 1.6 (0.74-3.63) | 0.226 | 3.4 | 0.7 (0.23-2.15) | 0.527 | 12.6 | 1.4 (0.80-2.49) | 0.241 | 11.5 | 1.0 (0.57-1.87) | 0.919 | 10.7 | 1.1 (0.61-2.16) | 0.670 | 5.7 | **3.2 (1.29-7.87)** | **0.009** |
| **Year of diagnosis** | 1976-1980 | 28.3 | Ref. |  | 4.0 | Ref. |  | 6.3 | Ref. |  | 8.0 | Ref. |  | 10.8 | Ref. |  | 13.9 | Ref. |  | 2.8 | Ref. |  |
|  | 1981-1985 | 27.4 | 1.0 (0.71-1.31) | 0.831 | 3.5 | 0.9 (0.34-2.25) | 0.779 | 5.9 | 1.0 (0.46-1.98) | 0.894 | 7.6 | 1.0 (0.50-1.83) | 0.902 | 12.5 | 1.2 (0.68-1.95) | 0.591 | 12.3 | 0.9 (0.55-1.44) | 0.622 | 1.4 | 0.5 (0.13-1.79) | 0.269 |
|  | 1986-1990 | 28.1 | 1.0 (0.75-1.32) | 0.957 | 5.0 | 1.3 (0.54-2.90) | 0.601 | 8.0 | 1.3 (0.66-2.48) | 0.462 | 9.2 | 1.2 (0.64-2.09) | 0.626 | 12.2 | 1.1 (0.69-1.86) | 0.632 | 11.6 | 0.8 (0.53-1.32) | 0.445 | 1.7 | 0.6 (0.20-1.91) | 0.396 |
|  | 1991-1995 | 23.6 | 0.8 (0.63-1.11) | 0.219 | 3.6 | 0.9 (0.38-2.12) | 0.809 | 3.4 | 0.5 (0.26-1.14) | 0.102 | 6.6 | 0.8 (0.45-1.51) | 0.535 | 9.2 | 0.8 (0.51-1.41) | 0.527 | 7.8 | **0.6 (0.35-0.91)** | **0.019** | 1.8 | 0.6 (0.21-1.85) | 0.398 |
|  | 1996-2000 | 27.4 | 1.0 (0.73-1.29) | 0.826 | 4.6 | 1.2 (0.50-2.72) | 0.728 | 5.6 | 0.9 (0.45-1.81) | 0.766 | 10.5 | 1.3 (0.74-2.34) | 0.347 | 11.4 | 1.1 (0.64-1.75) | 0.822 | 8.9 | 0.6 (0.39-1.04) | 0.072 | 2.2 | 0.8 (0.26-2.28) | 0.642 |
|  | 2001-2005 | 21.1 | 0.7 (0.54-1.03) | 0.076 | 3.3 | 0.8 (0.32-2.13) | 0.689 | 2.6 | 0.4 (0.17-1.03) | 0.051 | 8.9 | 1.1 (0.60-2.07) | 0.726 | 7.5 | 0.7 (0.39-1.25) | 0.223 | 6.4 | **0.5 (0.26-0.81)** | **0.006** | 2.3 | 0.8 (0.26-2.52) | 0.716 |
|  | 2006-2010 | 27.7 | 1.0 (0.71-1.35) | 0.888 | 4.6 | 1.2 (0.45-3.00) | 0.752 | 3.3 | 0.5 (0.21-1.32) | 0.163 | 10.2 | 1.3 (0.68-2.44) | 0.438 | 14.8 | 1.4 (0.81-2.34) | 0.239 | 7.7 | **0.6 (0.30-1.01)** | **0.048** | 0.9 | 0.3 (0.06-1.68) | 0.158 |
|  | 2011-2015 | 28.6 | 1.0 (0.73-1.39) | 0.957 | 4.2 | 1.1 (0.40-2.78) | 0.910 | 2.8 | 0.4 (0.17-1.19) | 0.097 | 12.7 | 1.6 (0.87-2.96) | 0.127 | 13.2 | 1.2 (0.71-2.11) | 0.468 | 7.0 | **0.5 (0.27-0.94)** | **0.027** | 3.3 | 1.2 (0.37-3.56) | 0.807 |
| **Age at study** | 15-24 years | 26.9 | Ref. |  | 4.1 | Ref. |  | 4.7 | Ref. |  | 10.2 | Ref. |  | 10.7 | Ref. |  | 9.4 | Ref. |  | 2.0 | Ref. |  |
|  | 25-34 years | 23.3 | 0.9 (0.74-1.01) | 0.066 | 3.0 | 0.7 (0.46-1.18) | 0.200 | 4.4 | 0.9 (0.63-1.41) | 0.767 | 7.1 | **0.7 (0.51-0.93)** | **0.014** | 10.2 | 1.0 (0.74-1.23) | 0.700 | 8.9 | 0.9 (0.71-1.24) | 0.668 | 1.6 | 0.8 (0.43-1.59) | 0.571 |
|  | 35-44 years | 26.5 | 1.0 (0.80-1.22) | 0.894 | 4.9 | 1.2 (0.68-2.12) | 0.536 | 5.6 | 1.2 (0.70-2.03) | 0.519 | 7.4 | 0.7 (0.46-1.12) | 0.142 | 13.4 | 1.2 (0.90-1.74) | 0.193 | 8.2 | 0.9 (0.57-1.33) | 0.523 | 2.8 | 1.4 (0.65-3.09) | 0.379 |
|  | 45-54 years | 41.2 | **1.5 (1.14-2.06)** | **0.010** | 12.3 | **3.0 (1.54-5.82)** | **0.001** | 8.2 | 1.7 (0.78-3.88) | 0.178 | 12.5 | 1.2 (0.65-2.30) | 0.539 | 19.2 | **1.8 (1.09-2.93)** | **0.026** | 13.0 | 1.4 (0.74-2.60) | 0.320 | 2.7 | 1.4 (0.33-5.68) | 0.657 |
|  | 55-64 years | 66.7 | 2.5 (1.11-5.54) | 0.121 | 0.0 | 0.0 (.-.) | 0.720 | 0.0 | 0.0 (.-.) | 0.700 | 0.0 | 0.0 (.-.) | 0.559 | 33.3 | 3.1 (0.62-15.49) | 0.208 | 33.3 | 3.5 (0.71-17.67) | 0.158 | 0.0 | 0.0 (.-.) | 0.805 |

Note: Bold font indicates statistically significant difference as compared to the reference group at p<0.05; Abbreviations: CI=confidence interval, CNS=central nervous system, Ref.=reference category

Supplemental Table 3. Incidence rates per 1000 person-years of specific musculoskeletal health conditions in adolescent and adult 5-year survivors of childhood and adolescent cancer stratified by diagnosis and age at diagnosis (only results of diagnostic groups with sample size >100 displayed).

|  |  | **Any musculoskeletal health condition** | **Osteoporosis** | **Arm- or leg-length discrepancy** | **Limited joint mobility** | **Persistent pain in bones or joints** | **Scoliosis** | **Changes to chest/ribs** |
| --- | --- | --- | --- | --- | --- | --- | --- | --- |
|  |  | Incidence rate (95% CI) | Incidence rate (95% CI) | Incidence rate (95% CI) | Incidence rate (95% CI) | Incidence rate (95% CI) | Incidence rate (95% CI) | Incidence rate (95% CI) |
| Leukaemia n=756 | 0-5 years at dx | 10.2 (7.88- 13.25) | 1.3 (0.64-2.55) | 0.2 (0.02-1.14) | 2.9 (1.82-4.58) | 4.3 (2.98-6.33) | 2.7 (1.64-4.36) | 0.6 (0.23-1.67) |
|  | 6-10 years at dx | 14.7 ( 10.69- 20.19) | 1.7 (0.70-4.02) | 1.0 (0.33-3.13) | 4.1 (2.31-7.15) | 5.9 (3.68-9.52) | 2.8 (1.40-5.61) | 0.0 (.-.) |
|  | 11-15 years at dx | **22.4 ( 16.33- 30.85)** | **8.1 (4.94- 13.16)** | 2.0 (0.74-5.23) | **9.2 (5.80- 14.62)** | **11.1 (7.21- 16.97)** | 2.0 (0.75-5.29) | 0.0 (.-.) |
|  | 16-20 years at dx | **27.5 ( 16.57- 45.59)** | **6.4 (2.39- 16.94)** | 1.5 (0.22- 10.96) | 6.1 (2.27- 16.13) | **24.0 ( 14.19- 40.45)** | 0.0 (.-.) | 0.0 (.-.) |
| Lymphoma  n=577 | 0-5 years at dx | 9.4 (4.70- 18.81) | 0.0 (.-.) | 2.1 (0.52-8.31) | 0.0 (.-.) | 5.1 (2.11- 12.16) | 1.1 (0.15-7.68) | 0.0 (.-.) |
|  | 6-10 years at dx | 12.2 (7.56- 19.57) | 1.2 (0.30-4.85) | 0.6 (0.09-4.31) | 2.4 (0.90-6.40) | 3.7 (1.66-8.22) | 4.6 (2.17-9.57) | 0.6 (0.09-4.34) |
|  | 11-15 years at dx | 14.2 ( 10.50- 19.09) | 1.7 (0.76-3.78) | 0.6 (0.14-2.26) | 3.8 (2.21-6.57) | **6.9 (4.56- 10.32)** | 2.6 (1.36-5.04) | 1.8 (0.79-3.91) |
|  | 16-20 years at dx | 10.9 (7.05- 16.94) | 2.2 (0.93-5.39) | 0.9 (0.22-3.58) | 0.9 (0.23-3.69) | 3.2 (1.52-6.71) | **1.5 (0.49-4.72)** | 2.3 (0.95-5.46) |
| CNS tumour n=417 | 0-5 years at dx | 15.3 (9.38- 24.99) | 0.8 (0.11-5.59) | 3.3 (1.24-8.80) | 4.9 (2.22- 11.00) | 3.2 (1.20-8.49) | 9.1 (4.92- 16.98) | 3.2 (1.19-8.45) |
|  | 6-10 years at dx | 14.3 (9.58- 21.32) | **2.9 (1.29-6.40)** | 2.4 (1.01-5.82) | 4.9 (2.63-9.08) | 2.8 (1.28-6.33) | 4.2 (2.12-8.48) | 0.0 (.-.) |
|  | 11-15 years at dx | **20.1 ( 14.03- 28.69)** | **3.4 (1.51-7.48)** | 0.6 (0.08-3.91) | **6.8 (3.85- 11.93)** | **8.1 (4.77- 13.61)** | 8.2 (4.85- 13.84) | 1.1 (0.27-4.30) |
|  | 16-20 years at dx | 5.4 (1.73- 16.61) | 3.4 (0.85- 13.57) | 0.0 (.-.) | 0.0 (.-.) | 1.7 (0.23- 11.81) | 0.0 (.-.) | 1.6 (0.23- 11.68) |
| Renal tumour n=121 | 0-5 years at dx | 15.4 ( 10.04- 23.61) | 1.2 (0.29-4.64) | 1.9 (0.61-5.86) | 2.4 (0.89-6.35) | 3.6 (1.60-7.93) | 8.8 (5.12- 15.18) | 0.6 (0.08-4.16) |
|  | 6-10 years at dx | 11.4 (3.67- 35.30) | 0.0 (.-.) | 6.6 (1.65- 26.40) | 3.4 (0.48- 24.32) | 0.0 (.-.) | 0.0 (.-.) | 0.0 (.-.) |
|  | 11-15 years at dx | 0.0 (.-.) | 0.0 (.-.) | 0.0 (.-.) | 0.0 (.-.) | 0.0 (.-.) | 0.0 (.-.) | 0.0 (.-.) |
|  | 16-20 years at dx | . (.-.) | 0.0 (.-.) | 0.0 (.-.) | 0.0 (.-.) | . (.-.) | 0.0 (.-.) | 0.0 (.-.) |
| Malignant bone tumour  n=139 | 0-5 years at dx | 48.3 ( 21.69-107.45) | 0.0 (.-.) | 19.7 (6.36- 61.10) | 16.5 (5.32- 51.16) | 15.8 (5.11- 49.11) | 11.3 (2.84- 45.35) | 0.0 (.-.) |
|  | 6-10 years at dx | **126.6 ( 85.54-187.34)** | 4.1 (1.02- 16.33) | **56.9 ( 34.87- 92.90)** | **66.5 ( 41.93-105.63)** | **31.3 ( 17.75- 55.04)** | **21.1 ( 10.54- 42.13)** | 0.0 (.-.) |
|  | 11-15 years at dx | **87.6 ( 62.87-121.96)** | 1.2 (0.17-8.53) | **21.3 ( 12.61- 35.94)** | **47.4 ( 32.00- 70.09)** | **20.3 ( 11.77- 34.91)** | **14.0 (7.53- 26.02)** | **3.7 (1.20- 11.56)** |
|  | 16-20 years at dx | **58.9 ( 32.60-106.30)** | 4.6 (1.14- 18.19) | **18.9 (9.02- 39.68)** | **29.2 ( 14.60- 58.36)** | **15.0 (6.22- 35.93)** | 0.0 (.-.) | 4.6 (1.16- 18.58) |
| Soft tissue sarcoma  n=166 | 0-5 years at dx | 10.1 (4.52- 22.41) | 0.0 (.-.) | 1.5 (0.21- 10.38) | 2.8 (0.69- 11.10) | 1.4 (0.20- 10.00) | 2.7 (0.68- 10.91) | 1.3 (0.19-9.36) |
|  | 6-10 years at dx | 21.2 (11.76- 38.34) | 0.0 (.-.) | **8.1 (3.38- 19.50)** | 5.0 (1.60- 15.35) | 1.5 (0.21- 10.34) | 8.4 (3.48- 20.10) | 3.1 (0.79- 12.56) |
|  | 11-15 years at dx | **23.4 (13.61- 40.38)** | 1.3 (0.18-9.21) | 2.7 (0.68- 10.82) | **12.1 (6.04- 24.13)** | 7.3 (3.02- 17.45) | 6.2 (2.32- 16.48) | 1.3 (0.18-9.23) |
|  | 16-20 years at dx | 14.3 (6.44- 31.91) | 0.0 (.-.) | 0.0 (.-.) | 4.6 (1.15- 18.46) | 6.4 (2.08- 19.96) | 2.1 (0.30- 15.25) | 0.0 (.-.) |
| Germ cell tumour n=151 | 0-5 years at dx | 9.6 (3.10- 29.81) | 0.0 (.-.) | 3.2 (0.45- 22.61) |  | 2.8 (0.39- 19.81) | 0.0 (.-.) | 0.0 (.-.) |
|  | 6-10 years at dx | 13.5 (4.35- 41.85) | **7.8 (1.95- 31.16)** | 0.0 (.-.) |  | 0.0 (.-.) | 3.7 (0.52- 26.35) | 0.0 (.-.) |
|  | 11-15 years at dx | 7.7 (2.49- 23.98) | 4.7 (1.17- 18.77) | 0.0 (.-.) |  | 2.4 (0.34- 16.91) | 0.0 (.-.) | 0.0 (.-.) |
|  | 16-20 years at dx | 9.9 (5.47- 17.82) | 2.5 (0.80-7.68) | 0.0 (.-.) |  | 5.8 (2.76- 12.16) | 2.5 (0.82-7.89) | 0.8 (0.11-5.71) |

Note: bold font indicates statistically significant difference as compared to the reference category (0-5 years at dx) at p<0.05. The diagnostic groups with sample size ≤100 are not displayed (neuroblastoma (n=87), retinoblastoma (n=45), hepatic tumour (n=15), other tumour (n=81), and Langerhans cell histiocytosis (n=90)). Abbreviations: CI=confidence interval, CNS=central nervous system, dx=diagnosis, n.a.=not applicable (as this diagnostic group had a sample size of <100)

Supplemental Table 4. Number of survivors with incidence of musculoskeletal health conditions, stratified by diagnosis and age at diagnosis (corresponding to the incidence rates in Supplemental Table 3).

|  |  | **Any musculoskeletal health condition** | **Osteoporosis** | **Arm- or leg-length discrepancy** | **Limited joint mobility** | **Persistent pain in bones or joints** | **Scoliosis** | **Changes to chest/ribs** |
| --- | --- | --- | --- | --- | --- | --- | --- | --- |
|  |  | Incidence: yes / Total category size | Incidence: yes / Total category size | Incidence: yes / Total category size | Incidence: yes / Total category size | Incidence: yes / Total category size | Incidence: yes / Total category size | Incidence: yes / Total category size |
| Leukaemia n=756 | 0-5 years at dx | 75/346 | 12/346 | 7/346 | 19/346 | 31/346 | 27/346 | 4/346 |
|  | 6-10 years at dx | 47/188 | 5/188 | 5/188 | 13/188 | 20/188 | 14/188 | 1/188 |
|  | 11-15 years at dx | 44/176 | 18/176 | 5/176 | 21/176 | 25/176 | 5/176 | 1/176 |
|  | 16-20 years at dx | 15/46 | 4/46 | 2/46 | 4/46 | 14/46 | 0/46 | 0/46 |
| Lymphoma  n=577 | 0-5 years at dx | 12/61 | 0/61 | 4/61 | 0/61 | 5/61 | 4/61 | 2/61 |
|  | 6-10 years at dx | 22/111 | 3/111 | 2/111 | 4/111 | 8/111 | 9/111 | 2/111 |
|  | 11-15 years at dx | 52/262 | 6/262 | 2/262 | 17/262 | 27/262 | 11/262 | 9/262 |
|  | 16-20 years at dx | 30/143 | 5/143 | 2/143 | 6/143 | 9/143 | 11/143 | 5/143 |
| CNS tumour n=417 | 0-5 years at dx | 19/76 | 4/76 | 6/76 | 8/76 | 7/76 | 14/76 | 5/76 |
|  | 6-10 years at dx | 36/145 | 7/145 | 8/145 | 13/145 | 7/145 | 13/145 | 0/145 |
|  | 11-15 years at dx | 37/152 | 9/152 | 3/152 | 15/152 | 18/152 | 16/152 | 3/152 |
|  | 16-20 years at dx | 4/44 | 2/44 | 0/44 | 0/44 | 2/44 | 2/44 | 1/44 |
| Renal tumour n=121 | 0-5 years at dx | 26/95 | 2/95 | 6/95 | 5/95 | 8/95 | 17/95 | 2/95 |
|  | 6-10 years at dx | 4/21 | 0/21 | 2/21 | 1/21 | 0/21 | 1/21 | 1/21 |
|  | 11-15 years at dx | 0/4 | 0/4 | 0/4 | 0/4 | 0/4 | 0/4 | 0/4 |
|  | 16-20 years at dx | 0/1 | 0/1 | 0/1 | 0/1 | 0/1 | 0/1 | 0/1 |
| Malignant bone tumour  n=139 | 0-5 years at dx | 6/10 | 0/10 | 3/10 | 3/10 | 3/10 | 2/10 | 0/10 |
|  | 6-10 years at dx | 26/38 | 2/38 | 16/38 | 19/38 | 13/38 | 10/38 | 0/38 |
|  | 11-15 years at dx | 37/63 | 1/63 | 15/63 | 28/63 | 16/63 | 10/63 | 3/63 |
|  | 16-20 years at dx | 16/28 | 2/28 | 8/28 | 12/28 | 9/28 | 0/28 | 2/28 |
| Soft tissue sarcoma  n=166 | 0-5 years at dx | 9/39 | 0/39 | 2/39 | 3/39 | 3/39 | 2/39 | 1/39 |
|  | 6-10 years at dx | 14/42 | 2/42 | 6/42 | 4/42 | 1/42 | 8/42 | 2/42 |
|  | 11-15 years at dx | 15/57 | 1/57 | 2/57 | 8/57 | 6/57 | 8/57 | 1/57 |
|  | 16-20 years at dx | 6/28 | 0/28 | 0/28 | 2/28 | 3/28 | 1/28 | 0/28 |
| Germ cell tumour n=151 | 0-5 years at dx | 4/21 | 0/21 | 2/21 | 1/21 | 1/21 | 0/21 | 0/21 |
|  | 6-10 years at dx | 4/16 | 2/16 | 0/16 | 1/16 | 1/16 | 1/16 | 0/16 |
|  | 11-15 years at dx | 4/37 | 2/37 | 0/37 | 0/37 | 2/37 | 1/37 | 0/37 |
|  | 16-20 years at dx | 11/77 | 3/77 | 0/77 | 1/77 | 7/77 | 4/77 | 2/77 |

Note: Only results of diagnoses with sample size >100 displayed. The diagnostic groups with sample size ≤100 are: neuroblastoma (n=87), retinoblastoma (n=45), hepatic tumour (n=15), other tumour (n=81), and Langerhans cell histiocytosis (n=90). Abbreviations: CNS=central nervous system, dx=diagnosis, n.a.=not applicable (as this diagnostic group had a sample size of <100)

Supplemental Figure 1. Frequency of various musculoskeletal health conditions by year since diagnosis at incidence in adolescent and adult 5-year survivors of childhood and adolescent cancer.

Appendix 1. Sensitivity analyses for imputation of missing values.

Sensitivity analysis 1 (“best case”): We imputed “no” for the missing values of the different musculoskeletal health condition (MSHC) variables (cumulative incidence and prevalence) and imputed the year of study participation for missing values in ‘incidence year’. The results are displayed in **Supplemental Tables 4a-4c**: Missing values before and after imputation are presented in **Supplemental Table 4a**. The prevalence and cumulative incidence proportions changed slightly (lower after imputation, e.g. prevalence of any MSHC from 21.2% to 20.2%; **Supplemental Table 4b**). Age at incidence and time since diagnosis at incidence were higher after imputation, as was to be expected based on the imputation of year of study participation as the incidence year (**Supplemental Table 4b**). The incidence rates and rate ratios were similar and the difference between groups did not change, e.g. “any MSHC”: incidence rate for stem cell transplantation from 25.8 to 24.9, and RR from 1.6 (95%CI: 1.0-2.5; **Table 3**) to RR 1.5 (95%CI: 1.0-2.2; **Supplemental Table 4c**).

Sensitivity analysis 2 (“median”): We imputed “no” for the missing values of the different musculoskeletal late effect variables (cumulative incidence and prevalence) if participants had answered at least one of the late effects questions (all organ systems). Participants who had missing values for the different musculoskeletal late effect variables remained coded as missing if they had missing information in all late effects questions (of all organ systems; n=129 (4.9%)). We additionally imputed missing values of “incidence year” with the median year of incidence stratified by musculoskeletal health condition.This resulted in a few survivors having an age at incidence that was <0, which we replaced with “0”, and a few survivors having an age at incidence lower than age at diagnosis, in which case we recoded the age at incidence to the age at diagnosis. The results are displayed in **Supplemental Table 5a-5c**: Missing values before and after imputation are presented in **Supplemental Table 5a**. The prevalence and cumulative incidence proportions were similar to the values before the imputation (**Supplemental Table 5b**). Age at incidence and time since diagnosis were slightly higher after imputation (e.g. overall age at incidence 16.8 years to 17.2 years; time since diagnosis at incidence overall from 7.3 years to 7.7 years; **Supplemental Table 5b**). The incidence rates and rate ratios were quite similar and the difference between groups did not change, e.g. “any MSHC”: incidence rate for stem cell transplantation from25.8 to 27.6, and RR from 1.6 (95%CI:1.0-2.5) to RR 1.5 (95%CI:1.0-2.2; **Supplemental Table 5c**).

Sensitivity analysis 3 (“worst case”): Based on the assumption that it is unlikely that participants who experienced a certain late effect did not answer the respectivequestion about this late effect, we imputed “no” for the missing values of the different musculoskeletal late effect variables (cumulative incidence and prevalence) if participants had answered at least one of the late effects questions (all organ systems). Participants who had missing values for the different musculoskeletal late effect variables remained coded as missing if they had missing information in all late effects questions (of all organ systems; n=129 (4.9%)), as it is possible that they experienced a late effect but simply felt it too tedious to fill in all the questions. We additionally imputed missing values of “incidence year” with the median year of incidence stratified by musculoskeletal health condition and year of diagnosis. This resulted in a few survivors having an age at incidence that was <0, which we replaced with “0”, and a few survivors having an age at incidence lower than age at diagnosis, in which case we recoded the age at incidence to the age at diagnosis. The results are displayed in **Supplemental Table 6a-6c**: Missing values before and after imputation are presented in **Supplemental Table 6a**. The prevalence and cumulative incidence proportions were similar to the values before the imputation (**Supplemental Table 6b**). Age at incidence and time since diagnosis at incidence were lower after imputation, as was expected (e.g. overall age at incidence 16.8 years to 16.6 years; time since diagnosis at incidence overall from 7.3 years to 7.1 years; **Supplemental Table 6b**). The incidence rates increased, as was to be expected, e.g. incidence rate of overall MSHC from 15.6 (95%CI: 14.3-17.0) to 18.4 (95%CI:17.0-19.8). The rate ratios were similar and the difference between groups did not change, e.g. “any MSHC”: incidence rate for females from 18.4 to 21.8, and RR from 1.4(95%CI: 1.2-1.7) to RR 1.4 (95%CI:1.2-1.6; **Supplemental Table 6c**). Only the diagnosis “germ cell tumour” was now statistically significantly associated with lower risk ([Ref. Leukemia] RR 0.6 (95%CI:0.4-1.0)), and SCT was no longer significantly associated with increased risk: SCT ([Ref. yes] RR 1.5 (95%CI:1.0-2.2)).

Supplemental Table 4a. Number of missings in outcomes of Table 2 before and after imputation (imputation - sensitivity analysis 1 “best case”).

|  | Prevalence | | Cumulative Incidence | | Condition no longer prevalent^a^ | | Age at incidence^b^ | | Time since diagnosis at incidence^c^ | |
| --- | --- | --- | --- | --- | --- | --- | --- | --- | --- | --- |
|  | Before n (%) | After n= (%) | Before n= (%) | After n= (%) | Before n= (%) | After n= (%) | Before n= (%) | After n= (%) | Before n= (%) | After n= (%) |
| Any musculoskeletal health condition | 129 (4.9) | 0 (0.0) | 190 (7.2) | 0 (0.0) | n.a. | n.a. | 125 (19.4) | 0 (0.0) | 125 (19.4) | 0 (0.0) |
| Osteoporosis | 129 (4.9) | 0 (0.0) | 130 (4.9) | 0 (0.0) | n.a. | n.a. | 19 (18.4) | 0 (0.0) | 19 (18.4) | 0 (0.0) |
| Arm- or leg-length discrepancy | 129 (4.9) | 0 (0.0) | 142 (5.4) | 0 (0.0) | n.a. | n.a. | 33 (27.3) | 0 (0.0) | 33 (27.3) | 0 (0.0) |
| Limited joint mobility | 129 (4.9) | 0 (0.0) | 135 (5.1) | 0 (0.0) | n.a. | n.a. | 36 (16.0) | 0 (0.0) | 36 (16.0) | 0 (0.0) |
| Persistent pain in bones or joints | 129 (4.9) | 0 (0.0) | 132 (5.0) | 0 (0.0) | n.a. | n.a. | 50 (17.9) | 0 (0.0) | 50 (17.9) | 0 (0.0) |
| Scoliosis | 129 (4.9) | 0 (0.0) | 166 (6.3) | 0 (0.0) | n.a. | n.a. | 73 (31.7) | 0 (0.0) | 73 (31.7) | 0 (0.0) |
| Changes to chest/ribs | 129 (4.9) | 0 (0.0) | 135 (5.1) | 0 (0.0) | n.a. | n.a. | 14 (28.0) | 0 (0.0) | 14 (28.0) | 0 (0.0) |

Abbreviations: n.a.=not applicable

^a^ Condition no longer prevalent: Number of survivors that indicated cumulative incidence 'yes' and prevalence 'no'. See missings listed under 'Prevalence' and 'Cumulative Incidence'.

^b^ Missing values in 'age at incidence' are only displayed for survivors who had cumulative incidence: yes (see **Table 2**)

^c^ Missing values in 'time since diagnosis at incidence' are only displayed for survivors who had cumulative incidence: yes (see **Table 2**)

Supplemental Table 4b. Prevalence and incidence of musculoskeletal health conditions before and after imputation in adolescent and adult 5-year survivors of childhood and adolescent cancer (N=2645; imputation - sensitivity analysis 1 “best case”).

|  | Prevalence | | Cumulative Incidence | | Condition no longer prevalent | | Age at incidence | | Time since diagnosis  at incidence^a^ | |
| --- | --- | --- | --- | --- | --- | --- | --- | --- | --- | --- |
|  |  | |  | |  | | in years | | in years | |
|  | Before n (%) | After n= (%) | Before n (%) | After n= (%) | Before  N=^b^ (%^c^) | After  N=^b^ (%^c^) | Before  Mean  (SD; range) | After  Mean  (SD; range) | Before  Mean  (SD; range) | After  Mean  (SD; range) |
| Any MSHC | 533 (21.2) | 533 (20.2) | 643 (26.2) | 643 (24.3) | 154 (24.0) | 154 (24.0) | 16.8 (8.47; 0-56) | 19.0 (9.56; 0-59) | 7.3 (7.53; 0-38) | 9.5 (8.83; 0-39) |
| Osteoporosis | 61 (2.4) | 61 (2.3) | 103 (4.1) | 103 (3.9) | 42 (40.8) | 42 (40.8) | 19.2 (9.62; 2-45) | 20.7 (9.65; 2-46) | 8.1 (8.20; 0-30) | 10.0 (8.91; 0-34) |
| Arm- or leg-length discrepancy | 107 (4.3) | 107 (4.0) | 121 (4.8) | 121 (4.6) | 23 (19.0) | 23 (19.0) | 14.1 (8.09; 0-43) | 17.6 (10.02; 0-46) | 5.2 (6.60; 0-32) | 9.4 (9.93; 0-37) |
| Limited joint mobility | 170 (6.8) | 170 (6.4) | 225 (9.0) | 225 (8.5) | 60 (26.7) | 60 (26.7) | 15.3 (6.96; 0-43) | 17.6 (9.07; 0-47) | 5.3 (6.55; 0-32) | 7.4 (8.29; 0-33) |
| Persistent pain in bones or joints | 188 (7.5) | 188 (7.1) | 280 (11.1) | 280 (10.6) | 93 (33.2) | 93 (33.2) | 20.0 (9.16; 1-56) | 21.6 (9.73; 1-56) | 9.8 (8.77; 0-38) | 11.3 (9.21; 0-38) |
| Scoliosis | 207 (8.2) | 207 (7.8) | 230 (9.3) | 230 (8.7) | 49 (21.3) | 49 (21.3) | 14.3 (7.06; 0-44) | 18.4 (9.59; 0-59) | 6.8 (6.37; 0-29) | 10.5 (8.82; 0-39) |
| Changes to chest/ribs | 41 (1.6) | 41 (1.6) | 50 (2.0) | 50 (1.9) | 13 (26.0) | 13 (26.0) | 17.0 (9.18; 6-50) | 19.5 (9.74; 6-50) | 6.1 (7.34; 0-33) | 9.2 (8.87; 0-33) |

Note: Proportions were calculated excluding missing values. Abbreviations: MSHC=musculoskeletal health conditions, n.a.=not applicable, SD=standard deviation
^a^ **Supplemental Figure 1** displays years since diagnosis at incidence for the specific musculoskeletal health condition

^b^ number of survivors that indicated cumulative incidence 'yes' and prevalence 'no'

^c^ number of survivors that indicated cumulative incidence 'yes' and prevalence 'no' divided by number of survivors that indicated cumulative incidence 'yes'

Supplemental Table 4c. Incidence rates (per 1000 person-years) and rate ratios of musculoskeletal health conditions in adolescent and adult 5-year survivors of childhood and adolescent cancer (N=2645; imputation - sensitivity analysis 1 “best case”).

|  | | Any musculoskeletal health condition | | | Osteoporosis | |  | Arm- or leg-length discrepancy | | | Limited joint mobility | | | Persistent pain in bones or joints | | | Scoliosis |  |  | Changes to chest/ribs | | |
| --- | --- | --- | --- | --- | --- | --- | --- | --- | --- | --- | --- | --- | --- | --- | --- | --- | --- | --- | --- | --- | --- | --- |
|  |  | Incidence rate | Rate ratios (95% CI) | p-value | Incidence rate | Rate ratios (95% CI) | p-value | Incidence rate | Rate ratios (95% CI) | p-value | Incidence rate | Rate ratios (95% CI) | p-value | Incidence rate | Rate ratios (95% CI) | p-value | Incidence rate | Rate ratios (95% CI) | p-value | Incidence rate | Rate ratios (95% CI) | p-value |
|  | Overall (95% CI) | 16.5 (15.3-17.9) | n.a. |  | 2.4 (2.0-2.9) | n.a. |  | 2.8 (2.4-3.4) | n.a. |  | 5.4 (4.7-6.1) | n.a. |  | 6.7 (5.9-7.5) | n.a. |  | 5.5 (4.8-6.3) | n.a. |  | 1.2 (0.9-1.5) | n.a. |  |
| Sex | Males | 13.8 | Ref. |  | 1.6 | Ref. |  | 2.3 | Ref. |  | 4.8 | Ref. |  | 5.3 | Ref. |  | 4.6 | Ref. |  | 1.3 | Ref. |  |
|  | Females | 19.8 | 1.4 (1.2-1.7) | **<0.001** | 3.3 | 2.1 (1.4-3.2) | **<0.001** | 3.5 | 1.5 (1.0-2.2) | **0.021** | 6.0 | 1.3 (1.0-1.6) | 0.088 | 8.2 | 1.5 (1.2-2.0) | **<0.001** | 6.5 | 1.4 (1.1-1.8) | **0.010** | 1.0 | 0.8 (0.4-1.4) | 0.343 |
| Diagnosis | Leukaemia | 15.2 | Ref. |  | 3.1 | Ref. |  | 1.5 | Ref. |  | 4.5 | Ref. |  | 7.2 | Ref. |  | 3.6 | Ref. |  | 0.5 | Ref. |  |
|  | Lymphoma | 14.0 | 0.9 (0.7-1.2) | 0.502 | 1.6 | 0.5 (0.3-1.0) | **0.026** | 1.1 | 0.8 (0.3-1.7) | 0.483 | 3.0 | 0.7 (0.4-1.1) | 0.086 | 5.6 | 0.8 (0.5-1.1) | 0.145 | 4.0 | 1.1 (0.7-1.8) | 0.658 | 2.0 | 4.4 (1.7-13.4) | **0.001** |
|  | CNS tumour | 16.9 | 1.1 (0.9-1.4) | 0.377 | 3.6 | 1.2 (0.7-2.0) | 0.559 | 2.8 | 1.9 (0.9-3.8) | 0.067 | 5.9 | 1.3 (0.8-2.0) | 0.217 | 5.5 | 0.8 (0.5-1.1) | 0.182 | 7.6 | 2.1 (1.4-3.2) | **<0.001** | 1.4 | 3.1 (1.0-10.6) | **0.033** |
|  | Neuroblastoma | 15.1 | 1.0 (0.6-1.5) | 0.986 | 1.2 | 0.4 (0.0-1.5) | 0.150 | 3.5 | 2.4 (0.8-6.2) | 0.086 | 3.5 | 0.8 (0.3-1.8) | 0.594 | 4.8 | 0.7 (0.3-1.4) | 0.273 | 11.3 | 3.1 (1.7-5.5) | **<0.001** | 0.6 | 1.2 (0.0-10.1) | 0.790 |
|  | Retinoblastoma | 9.5 | 0.6 (0.3-1.3) | 0.183 | 0.0 | 0.0 (0.0-1.4) | 0.066 | 2.2 | 1.5 (0.2-6.2) | 0.561 | 3.4 | 0.8 (0.2-2.3) | 0.696 | 5.7 | 0.8 (0.3-1.9) | 0.646 | 4.5 | 1.2 (0.3-3.4) | 0.643 | 0.0 | 0.0 (0.0-12.0) | 0.663 |
|  | Renal tumour | 15.1 | 1.0 (0.7-1.5) | 0.997 | 0.9 | 0.3 (0.0-1.1) | 0.055 | 3.7 | 2.5 (0.9-5.9) | **0.045** | 2.7 | 0.6 (0.2-1.4) | 0.238 | 3.6 | 0.5 (0.2-1.0) | **0.044** | 8.6 | 2.4 (1.3-4.2) | **0.003** | 1.3 | 2.9 (0.5-13.5) | 0.167 |
|  | Hepatic tumour | 7.9 | 0.5 (0.1-1.9) | 0.373 | 0.0 | 0.0 (0.0-4.8) | 0.453 | 0.0 | 0.0 (0.0-10.5) | 0.682 | 3.9 | 0.9 (0.0-5.0) | 0.982 | 3.9 | 0.5 (0.0-3.1) | 0.613 | 0.0 | 0.0 (0.0-4.1) | 0.393 | 0.0 | 0.0 (0.0-42.1) | 0.887 |
|  | Malignant bone tumour | 71.2 | 4.7 (3.6-6.1) | **<0.001** | 2.5 | 0.8 (0.2-2.1) | 0.699 | 26.8 | 18.1 (10.3-33.0) | **<0.001** | 41.7 | 9.2 (6.3-13.5) | **<0.001** | 23.7 | 3.3 (2.2-4.8) | **<0.001** | 12.3 | 3.4 (1.9-5.8) | **<0.001** | 2.5 | 5.4 (1.3-21.3) | **0.011** |
|  | Soft tissue sarcoma | 18.1 | 1.2 (0.8-1.7) | 0.293 | 1.1 | 0.3 (0.1-1.1) | 0.052 | 3.7 | 2.5 (1.0-5.7) | **0.027** | 6.5 | 1.4 (0.8-2.5) | 0.189 | 4.7 | 0.7 (0.3-1.2) | 0.144 | 7.0 | 1.9 (1.1-3.4) | **0.020** | 1.4 | 3.1 (0.6-13.2) | 0.101 |
|  | Germ cell tumour | 10.0 | 0.7 (0.4-1.0) | 0.053 | 2.9 | 1.0 (0.4-2.2) | 0.954 | 0.8 | 0.6 (0.1-2.3) | 0.451 | 1.2 | 0.3 (0.1-0.8) | **0.010** | 4.6 | 0.6 (0.3-1.2) | 0.143 | 2.5 | 0.7 (0.2-1.6) | 0.410 | 0.8 | 1.7 (0.2-9.8) | 0.499 |
|  | Other tumour | 14.9 | 1.0 (0.6-1.6) | 0.983 | 4.5 | 1.5 (0.4-3.7) | 0.426 | 2.6 | 1.7 (0.3-5.9) | 0.381 | 1.7 | 0.4 (0.0-1.5) | 0.156 | 5.2 | 0.7 (0.3-1.6) | 0.472 | 6.1 | 1.7 (0.6-3.8) | 0.212 | 0.9 | 1.9 (0.0-15.5) | 0.555 |
|  | Langerhans cell histiocytosis | 14.0 | 0.9 (0.5-1.5) | 0.770 | 2.7 | 0.9 (0.2-2.4) | 0.843 | 1.3 | 0.9 (0.1-3.7) | 0.956 | 3.4 | 0.7 (0.2-1.8) | 0.554 | 9.7 | 1.3 (0.7-2.4) | 0.301 | 6.9 | 1.9 (0.9-3.9) | 0.078 | 0.7 | 1.5 (0.0-12.0) | 0.690 |
| Relapse | No | 15.3 | Ref. |  | 2.1 | Ref. |  | 2.9 | Ref. |  | 4.7 | Ref. |  | 6.2 | Ref. |  | 5.0 | Ref. |  | 1.1 | Ref. |  |
|  | Yes | 21.6 | 1.4 (1.2-1.7) | **<0.001** | 3.5 | 1.7 (1.0-2.6) | **0.024** | 2.8 | 1.0 (0.6-1.6) | 0.995 | 8.2 | 1.8 (1.3-2.4) | **<0.001** | 8.5 | 1.4 (1.0-1.8) | **0.020** | 7.4 | 1.5 (1.1-2.0) | **0.010** | 1.6 | 1.5 (0.7-2.8) | 0.219 |
| Age at diagnosis | 0-5 years at dx | 12.4 | Ref. |  | 1.4 | Ref. |  | 2.4 | Ref. |  | 3.1 | Ref. |  | 4.5 | Ref. |  | 5.9 | Ref. |  | 1.0 | Ref. |  |
|  | 6-10 years at dx | 18.9 | 1.5 (1.2-1.9) | **<0.001** | 2.2 | 1.6 (0.8-3.0) | 0.139 | 4.2 | 1.7 (1.1-2.8) | **0.016** | 6.0 | 1.9 (1.3-2.9) | **0.001** | 6.0 | 1.3 (0.9-1.9) | 0.119 | 6.3 | 1.1 (0.8-1.5) | 0.720 | 0.6 | 0.6 (0.2-1.7) | 0.360 |
|  | 11-15 years at dx | 21.9 | 1.8 (1.4-2.2) | **<0.001** | 3.7 | 2.6 (1.5-4.6) | **<0.001** | 2.8 | 1.2 (0.7-1.9) | 0.538 | 9.1 | 2.9 (2.0-4.2) | **<0.001** | 9.9 | 2.2 (1.6-3.0) | **<0.001** | 5.5 | 0.9 (0.7-1.3) | 0.636 | 1.6 | 1.6 (0.8-3.4) | 0.182 |
|  | 16-20 years at dx | 15.2 | 1.2 (0.9-1.6) | 0.124 | 3.0 | 2.1 (1.1-4.1) | **0.018** | 2.0 | 0.8 (0.4-1.6) | 0.595 | 4.1 | 1.3 (0.8-2.1) | 0.267 | 7.8 | 1.7 (1.2-2.5) | **0.004** | 3.3 | 0.6 (0.3-0.9) | **0.010** | 1.7 | 1.8 (0.7-4.0) | 0.163 |
| Surgery | No | 14.9 | Ref. |  | 3.1 | Ref. |  | 1.3 | Ref. |  | 4.1 | Ref. |  | 7.0 | Ref. |  | 3.7 | Ref. |  | 0.5 | Ref. |  |
|  | Yes | 17.8 | 1.2 (1.0-1.4) | **0.045** | 2.2 | 0.7 (0.5-1.1) | 0.079 | 3.6 | 2.7 (1.6-4.8) | **<0.001** | 6.2 | 1.5 (1.1-2.1) | **0.006** | 6.6 | 0.9 (0.7-1.2) | 0.594 | 6.4 | 1.7 (1.3-2.4) | **<0.001** | 1.4 | 2.7 (1.2-7.2) | **0.008** |
| Chemotherapy | No | 13.8 | Ref. |  | 2.1 | Ref. |  | 2.5 | Ref. |  | 4.0 | Ref. |  | 5.3 | Ref. |  | 6.7 | Ref. |  | 0.7 | Ref. |  |
|  | Yes | 17.9 | 1.3 (1.1-1.6) | **0.012** | 2.6 | 1.2 (0.7-2.2) | 0.412 | 3.0 | 1.2 (0.8-2.1) | 0.411 | 6.0 | 1.5 (1.0-2.2) | **0.028** | 7.2 | 1.3 (1.0-1.9) | 0.063 | 5.3 | 0.8 (0.6-1.1) | 0.129 | 1.3 | 1.8 (0.8-5.2) | 0.166 |
| Radiotherapy | No | 17.2 | Ref. |  | 2.6 | Ref. |  | 2.8 | Ref. |  | 5.8 | Ref. |  | 7.2 | Ref. |  | 5.0 | Ref. |  | 0.9 | Ref. |  |
|  | Yes | 16.3 | 0.9 (0.8-1.1) | 0.536 | 2.5 | 1.0 (0.6-1.5) | 0.882 | 2.8 | 1.0 (0.7-1.5) | 0.938 | 4.8 | 0.8 (0.6-1.1) | 0.187 | 5.9 | 0.8 (0.6-1.1) | 0.120 | 6.7 | 1.4 (1.0-1.8) | **0.026** | 1.5 | 1.6 (0.9-3.1) | 0.095 |
| SCT | No | 16.7 | Ref. |  | 2.5 | Ref. |  | 2.9 | Ref. |  | 5.4 | Ref. |  | 6.7 | Ref. |  | 5.5 | Ref. |  | 1.0 | Ref. |  |
|  | Yes | 24.9 | 1.5 (1.0-2.2) | **0.047** | 4.8 | 2.0 (0.7-4.4) | 0.137 | 2.4 | 0.8 (0.2-2.5) | 0.820 | 9.0 | 1.7 (0.8-3.1) | 0.111 | 8.2 | 1.2 (0.6-2.3) | 0.498 | 7.4 | 1.3 (0.6-2.6) | 0.398 | 4.0 | 3.8 (1.2-9.7) | **0.016** |
| Year of diagnosis | 1970-1980 | 9.6 | Ref. |  | 1.2 | Ref. |  | 1.9 | Ref. |  | 2.5 | Ref. |  | 3.3 | Ref. |  | 4.4 | Ref. |  | 0.9 | Ref. |  |
|  | 1981-1985 | 10.7 | 1.1 (0.8-1.6) | 0.545 | 1.3 | 1.0 (0.4-3.2) | 0.945 | 2.2 | 1.1 (0.5-2.7) | 0.750 | 2.9 | 1.1 (0.6-2.4) | 0.714 | 4.7 | 1.4 (0.8-2.6) | 0.232 | 4.6 | 1.0 (0.6-1.8) | 0.873 | 0.5 | 0.6 (0.1-2.7) | 0.433 |
|  | 1986-1990 | 14.1 | 1.5 (1.0-2.1) | **0.021** | 2.3 | 1.9 (0.8-5.3) | 0.143 | 3.8 | 2.0 (1.0-4.3) | **0.047** | 4.4 | 1.7 (0.9-3.5) | 0.072 | 5.7 | 1.7 (1.0-3.1) | **0.044** | 5.5 | 1.2 (0.7-2.1) | 0.386 | 0.8 | 0.9 (0.2-3.6) | 0.858 |
|  | 1991-1995 | 14.4 | 1.5 (1.1-2.1) | **0.014** | 2.1 | 1.7 (0.7-4.8) | 0.233 | 2.0 | 1.0 (0.5-2.4) | 0.965 | 3.8 | 1.5 (0.8-3.1) | 0.177 | 5.4 | 1.6 (0.9-2.9) | 0.077 | 4.6 | 1.0 (0.6-1.8) | 0.899 | 1.0 | 1.2 (0.4-4.5) | 0.796 |
|  | 1996-2000 | 22.9 | 2.4 (1.7-3.4) | **<0.001** | 3.5 | 2.9 (1.2-8.2) | **0.012** | 4.3 | 2.2 (1.0-5.1) | **0.025** | 8.3 | 3.3 (1.8-6.5) | **<0.001** | 9.0 | 2.7 (1.5-4.9) | **<0.001** | 6.8 | 1.5 (0.9-2.7) | 0.097 | 1.7 | 1.9 (0.6-7.2) | 0.257 |
|  | 2001-2005 | 23.9 | 2.5 (1.7-3.7) | **<0.001** | 3.5 | 2.9 (1.0-8.8) | **0.035** | 2.8 | 1.4 (0.5-3.9) | 0.444 | 9.7 | 3.9 (2.0-8.0) | **<0.001** | 8.1 | 2.4 (1.3-4.7) | **0.005** | 6.6 | 1.5 (0.8-2.8) | 0.198 | 2.4 | 2.8 (0.8-11.0) | 0.090 |
|  | 2006-2010 | 34.6 | 3.6 (2.4-5.4) | **<0.001** | 5.4 | 4.4 (1.5-13.6) | **0.003** | 3.8 | 2.0 (0.6-5.5) | 0.180 | 12.2 | 4.8 (2.4-10.2) | **<0.001** | 18.2 | 5.4 (3.0-10.2) | **<0.001** | 8.5 | 1.9 (1.0-3.8) | **0.046** | 1.1 | 1.2 (0.1-7.4) | 0.794 |
|  | 2011-2015 | 40.4 | 4.2 (2.8-6.3) | **<0.001** | 5.2 | 4.3 (1.4-13.5) | **0.005** | 3.4 | 1.8 (0.5-5.3) | 0.268 | 16.4 | 6.5 (3.3-13.5) | **<0.001** | 16.9 | 5.1 (2.7-9.6) | **<0.001** | 8.8 | 2.0 (1.0-4.0) | **0.043** | 4.0 | 4.6 (1.2-18.2) | **0.012** |
| Age at study | 15-24 years | 22.6 | Ref. |  | 3.2 | Ref. |  | 3.7 | Ref. |  | 8.2 | Ref. |  | 8.5 | Ref. |  | 7.4 | Ref. |  | 1.5 | Ref. |  |
|  | 25-34 years | 12.6 | 0.6 (0.5-0.7) | **<0.001** | 1.5 | 0.5 (0.3-0.8) | **0.002** | 2.3 | 0.6 (0.4-1.0) | **0.021** | 3.7 | 0.5 (0.3-0.6) | **<0.001** | 5.3 | 0.6 (0.5-0.8) | **0.001** | 4.6 | 0.6 (0.5-0.8) | **0.001** | 0.8 | 0.5 (0.3-1.1) | 0.070 |
|  | 35-44 years | 11.2 | 0.5 (0.4-0.6) | **<0.001** | 1.9 | 0.6 (0.3-1.1) | 0.074 | 2.2 | 0.6 (0.3-1.0) | 0.060 | 2.9 | 0.4 (0.2-0.6) | **<0.001** | 5.3 | 0.6 (0.4-0.9) | **0.008** | 3.2 | 0.4 (0.3-0.7) | **<0.001** | 1.1 | 0.7 (0.3-1.6) | 0.410 |
|  | 45-54 years | 14.1 | 0.6 (0.4-0.9) | **0.011** | 4.0 | 1.3 (0.5-2.6) | 0.509 | 2.6 | 0.7 (0.3-1.6) | 0.449 | 4.0 | 0.5 (0.2-1.0) | **0.027** | 6.2 | 0.7 (0.4-1.3) | 0.260 | 4.2 | 0.6 (0.3-1.1) | 0.085 | 0.9 | 0.6 (0.1-2.3) | 0.482 |
|  | 55-64 years | 18.0 | 0.8 (0.1-2.9) | 0.824 | 0.0 | 0.0 (0.0-10.7) | 0.701 | 0.0 | 0.0 (0.0-9.3) | 0.664 | 0.0 | 0.0 (0.0-4.1) | 0.403 | 9.0 | 1.1 (0.0-6.0) | 0.858 | 9.0 | 1.2 (0.0-6.9) | 0.765 | 0.0 | 0.0 (0.0-23.3) | 0.844 |

Note: Bold font indicates statistically significant difference as compared to the reference group at p<0.05; Abbreviations: CI=confidence interval, CNS=central nervous system, Ref.=reference category, SCT=stem cell therapy

Supplemental Table 5a. Number of missings in outcomes of Table 2 before and after imputation (imputation - sensitivity analysis 2 “median”).

|  | Prevalence | | Cumulative Incidence | | Condition no longer prevalent^a^ | | Age at incidence^b^ | | Time since diagnosis at incidence^c^ | |
| --- | --- | --- | --- | --- | --- | --- | --- | --- | --- | --- |
|  | Before n (%) | After n= (%) | Before n= (%) | After n= (%) | Before n= (%) | After n= (%) | Before n= (%) | After n= (%) | Before n= (%) | After n= (%) |
| Any musculoskeletal health condition | 129 (4.9) | 129 (4.9) | 190 (7.2) | 129 (4.9) | n.a. | n.a. | 125 (19.4) | 0 (0.0) | 125 (19.4) | 0 (0.0) |
| Osteoporosis | 129 (4.9) | 129 (4.9) | 130 (4.9) | 129 (4.9) | n.a. | n.a. | 19 (18.4) | 0 (0.0) | 19 (18.4) | 0 (0.0) |
| Arm- or leg-length discrepancy | 129 (4.9) | 129 (4.9) | 142 (5.4) | 129 (4.9) | n.a. | n.a. | 33 (27.3) | 0 (0.0) | 33 (27.3) | 0 (0.0) |
| Limited joint mobility | 129 (4.9) | 129 (4.9) | 135 (5.1) | 129 (4.9) | n.a. | n.a. | 36 (16.0) | 0 (0.0) | 36 (16.0) | 0 (0.0) |
| Persistent pain in bones or joints | 129 (4.9) | 129 (4.9) | 132 (5.0) | 129 (4.9) | n.a. | n.a. | 50 (17.9) | 0 (0.0) | 50 (17.9) | 0 (0.0) |
| Scoliosis | 129 (4.9) | 129 (4.9) | 166 (6.3) | 129 (4.9) | n.a. | n.a. | 73 (31.7) | 0 (0.0) | 73 (31.7) | 0 (0.0) |
| Changes to chest/ribs | 129 (4.9) | 129 (4.9) | 135 (5.1) | 129 (4.9) | n.a. | n.a. | 14 (28.0) | 0 (0.0) | 14 (28.0) | 0 (0.0) |

Abbreviations: n.a.=not applicable

^a^ Condition no longer prevalent: Number of survivors that indicated cumulative incidence 'yes' and prevalence 'no'. See missings listed under 'Prevalence' and 'Cumulative Incidence'.

^b^ Missing values in 'age at incidence' are only displayed for survivors who had cumulative incidence: yes (see **Table 2**)

^c^ Missing values in 'time since diagnosis at incidence' are only displayed for survivors who had cumulative incidence: yes (see **Table 2**)

Supplemental Table 5b. Prevalence and incidence of musculoskeletal health conditions in adolescent and adult 5-year survivors of childhood and adolescent cancer (N=2645; imputation - sensitivity analysis 2 “median”).

|  | Prevalence | | Cumulative Incidence | | Condition no longer prevalent | | Age at incidence | | Time since diagnosis  at incidence^a^ | |
| --- | --- | --- | --- | --- | --- | --- | --- | --- | --- | --- |
|  |  | |  | |  | | in years | | in years | |
|  | Before n (%) | After n= (%) | Before n (%) | After n= (%) | Before  N=^b^ (%^c^) | After  N=^b^ (%^c^) | Before  Mean  (SD; range) | After  Mean  (SD; range) | Before  Mean  (SD; range) | After  Mean  (SD; range) |
| Any MSHC | 533 (21.2) | 533 (21.2) | 643 (26.2) | 643 (25.6) | 154 (24.0) | 154 (24.0) | 16.8 (8.47; 0-56) | 17.2 (8.52; 0-56) | 7.3 (7.53; 0-38) | 7.7 (7.67; 0-38) |
| Osteoporosis | 61 (2.4) | 61 (2.4) | 103 (4.1) | 103 (4.1) | 42 (40.8) | 42 (40.8) | 19.2 (9.62; 2-45) | 19.4 (9.14; 2-45) | 8.1 (8.20; 0-30) | 8.7 (8.14; 0-30) |
| Arm- or leg-length discrepancy | 107 (4.3) | 107 (4.3) | 121 (4.8) | 121 (4.8) | 23 (19.0) | 23 (19.0) | 14.1 (8.09; 0-43) | 14.6 (8.01; 0-43) | 5.2 (6.60; 0-32) | 6.3 (7.08; 0-32) |
| Limited joint mobility | 170 (6.8) | 170 (6.8) | 225 (9.0) | 225 (8.9) | 60 (26.7) | 60 (26.7) | 15.3 (6.96; 0-43) | 16.4 (7.66; 0-43) | 5.3 (6.55; 0-32) | 6.1 (7.11; 0-32) |
| Persistent pain in bones or joints | 188 (7.5) | 188 (7.5) | 280 (11.1) | 280 (11.1) | 93 (33.2) | 93 (33.2) | 20.0 (9.16; 1-56) | 20.6 (9.15; 1-56) | 9.8 (8.77; 0-38) | 10.4 (8.89; 0-38) |
| Scoliosis | 207 (8.2) | 207 (8.2) | 230 (9.3) | 230 (9.1) | 49 (21.3) | 49 (21.3) | 14.3 (7.06; 0-44) | 15.0 (7.41; 0-44) | 6.8 (6.37; 0-29) | 7.1 (6.83; 0-29) |
| Changes to chest/ribs | 41 (1.6) | 41 (1.6) | 50 (2.0) | 50 (2.0) | 13 (26.0) | 13 (26.0) | 17.0 (9.18; 6-50) | 17.8 (9.20; 6-50) | 6.1 (7.34; 0-33) | 7.6 (8.01; 0-33) |

Note: Proportions were calculated excluding missing values. Abbreviations: MSHC=musculoskeletal health conditions, n.a.=not applicable, SD=standard deviation
^a^ **Supplemental Figure 1** displays years since diagnosis at incidence for the specific musculoskeletal health condition

^b^ number of survivors that indicated cumulative incidence 'yes' and prevalence 'no'

^c^ number of survivors that indicated cumulative incidence 'yes' and prevalence 'no' divided by number of survivors that indicated cumulative incidence 'yes'

Supplemental Table 5c. Incidence rates (per 1000 person-years) and rate ratios of musculoskeletal health conditions in adolescent and adult 5-year survivors of childhood and adolescent cancer (N=2645; imputation - sensitivity analysis 2 “median”).

|  | | Any musculoskeletal health condition | | | Osteoporosis | |  | Arm- or leg-length discrepancy | | | Limited joint mobility | | | Persistent pain in bones or joints | | | Scoliosis |  |  | Changes to chest/ribs | | |
| --- | --- | --- | --- | --- | --- | --- | --- | --- | --- | --- | --- | --- | --- | --- | --- | --- | --- | --- | --- | --- | --- | --- |
|  |  | Incidence rate | Rate ratios (95% CI)a | p-value | Incidence rate | Rate ratios (95% CI)a | p-value | Incidence rate | Rate ratios (95% CI)a | p-value | Incidence rate | Rate ratios (95% CI)a | p-value | Incidence rate | Rate ratios (95% CI)a | p-value | Incidence rate | Rate ratios (95% CI)a | p-value | Incidence rate | Rate ratios (95% CI)a | p-value |
|  | Overall (95% CI) | 18.2 (16.8-19.6) | n.a. |  | 2.5 (2.1-3.1) | n.a. |  | 3.0 (2.5-3.6) | n.a. |  | 5.7 (5.0-6.5) | n.a. |  | 7.1 (6.3-8.0) | n.a. |  | 5.9 (5.2-6.8) | n.a. |  | 1.2 (0.9-1.6) | n.a. |  |
| Sex | Males | 15.3 | Ref. |  | 1.7 | Ref. |  | 2.5 | Ref. |  | 5.2 | Ref. |  | 5.7 | Ref. |  | 5.0 | Ref. |  | 1.4 | Ref. |  |
|  | Females | 21.5 | 1.4 (1.2-1.7) | **<0.001** | 3.5 | 2.0 (1.3-3.1) | **0.001** | 3.7 | 1.5 (1.0-2.2) | **0.030** | 6.3 | 1.2 (0.9-1.6) | 0.137 | 8.6 | 1.5 (1.2-1.9) | **0.001** | 7.0 | 1.4 (1.1-1.8) | **0.014** | 1.0 | 0.7 (0.4-1.3) | 0.289 |
| Diagnosis | Leukaemia | 16.6 | Ref. |  | 3.3 | Ref. |  | 1.6 | Ref. |  | 4.8 | Ref. |  | 7.7 | Ref. |  | 3.9 | Ref. |  | 0.5 | Ref. |  |
|  | Lymphoma | 15.4 | 0.9 (0.7-1.2) | 0.534 | 1.7 | 0.5 (0.3-1.0) | **0.025** | 1.2 | 0.8 (0.3-1.7) | 0.476 | 3.3 | 0.7 (0.4-1.1) | 0.091 | 5.9 | 0.8 (0.5-1.1) | 0.147 | 4.3 | 1.1 (0.7-1.8) | 0.639 | 2.2 | 4.4 (1.7-13.5) | **0.001** |
|  | CNS tumour | 18.6 | 1.1 (0.9-1.4) | 0.362 | 3.8 | 1.2 (0.7-2.0) | 0.571 | 2.9 | 1.9 (0.9-3.8) | 0.069 | 6.2 | 1.3 (0.8-2.0) | 0.225 | 5.8 | 0.8 (0.5-1.1) | 0.169 | 8.2 | 2.1 (1.4-3.3) | **<0.001** | 1.5 | 3.1 (1.0-10.5) | **0.035** |
|  | Neuroblastoma | 16.7 | 1.0 (0.6-1.6) | 0.945 | 1.2 | 0.4 (0.0-1.5) | 0.150 | 3.8 | 2.4 (0.8-6.3) | 0.083 | 3.8 | 0.8 (0.3-1.8) | 0.604 | 5.2 | 0.7 (0.3-1.4) | 0.278 | 12.4 | 3.2 (1.7-5.6) | **<0.001** | 0.6 | 1.2 (0.0-10.2) | 0.786 |
|  | Retinoblastoma | 10.4 | 0.6 (0.3-1.3) | 0.183 | 0.0 | 0.0 (0.0-1.4) | 0.069 | 2.4 | 1.5 (0.2-6.3) | 0.550 | 3.7 | 0.8 (0.2-2.4) | 0.725 | 6.2 | 0.8 (0.3-2.0) | 0.683 | 5.0 | 1.3 (0.3-3.5) | 0.605 | 0.0 | 0.0 (0.0-12.2) | 0.668 |
|  | Renal tumour | 16.5 | 1.0 (0.7-1.5) | 0.984 | 0.9 | 0.3 (0.0-1.1) | 0.053 | 3.9 | 2.5 (0.9-6.0) | **0.043** | 2.9 | 0.6 (0.2-1.4) | 0.233 | 3.8 | 0.5 (0.2-1.0) | **0.041** | 9.4 | 2.4 (1.3-4.2) | **0.003** | 1.4 | 2.9 (0.5-13.5) | 0.168 |
|  | Hepatic tumour | 7.9 | 0.5 (0.1-1.8) | 0.296 | 0.0 | 0.0 (0.0-4.5) | 0.431 | 0.0 | 0.0 (0.0-9.9) | 0.665 | 3.9 | 0.8 (0.0-4.7) | 0.933 | 3.9 | 0.5 (0.0-2.9) | 0.561 | 0.0 | 0.0 (0.0-3.8) | 0.367 | 0.0 | 0.0 (0.0-39.7) | 0.881 |
|  | Malignant bone tumour | 80.3 | 4.8 (3.7-6.3) | **<0.001** | 2.5 | 0.8 (0.2-2.0) | 0.639 | 28.1 | 17.8 (10.1-32.4) | **<0.001** | 45.3 | 9.4 (6.5-13.8) | **<0.001** | 25.2 | 3.3 (2.2-4.8) | **<0.001** | 12.7 | 3.3 (1.9-5.6) | **<0.001** | 2.6 | 5.2 (1.3-20.6) | **0.013** |
|  | Soft tissue sarcoma | 19.7 | 1.2 (0.8-1.7) | 0.305 | 1.1 | 0.3 (0.1-1.1) | **0.048** | 3.9 | 2.5 (1.0-5.6) | **0.028** | 6.9 | 1.4 (0.8-2.5) | 0.199 | 5.0 | 0.6 (0.3-1.2) | 0.133 | 7.6 | 2.0 (1.1-3.4) | **0.019** | 1.5 | 3.1 (0.6-13.0) | 0.105 |
|  | Germ cell tumour | 10.7 | 0.6 (0.4-1.0) | **0.042** | 3.1 | 1.0 (0.4-2.2) | 0.944 | 0.9 | 0.5 (0.1-2.3) | 0.445 | 1.3 | 0.3 (0.1-0.8) | **0.009** | 4.8 | 0.6 (0.3-1.2) | 0.137 | 2.7 | 0.7 (0.2-1.6) | 0.400 | 0.9 | 1.7 (0.2-9.8) | 0.499 |
|  | Other tumour | 16.8 | 1.0 (0.6-1.7) | 0.920 | 4.8 | 1.5 (0.4-3.7) | 0.420 | 2.7 | 1.7 (0.3-5.9) | 0.380 | 1.8 | 0.4 (0.0-1.4) | 0.153 | 5.6 | 0.7 (0.3-1.6) | 0.470 | 6.7 | 1.7 (0.7-3.9) | 0.191 | 0.9 | 1.9 (0.0-15.3) | 0.560 |
|  | Langerhans cell histiocytosis | 16.1 | 1.0 (0.6-1.6) | 0.932 | 3.0 | 0.9 (0.2-2.5) | 0.904 | 1.5 | 0.9 (0.1-3.9) | 0.998 | 3.7 | 0.8 (0.2-1.9) | 0.625 | 10.9 | 1.4 (0.7-2.5) | 0.229 | 7.7 | 2.0 (0.9-4.0) | 0.064 | 0.7 | 1.5 (0.0-12.5) | 0.667 |
| Relapse | No | 16.7 | Ref. |  | 2.2 | Ref. |  | 3.0 | Ref. |  | 4.9 | Ref. |  | 6.5 | Ref. |  | 5.4 | Ref. |  | 1.1 | Ref. |  |
|  | Yes | 24.3 | 1.5 (1.2-1.7) | **<0.001** | 3.8 | 1.7 (1.1-2.6) | **0.017** | 3.1 | 1.0 (0.6-1.6) | 0.929 | 9.0 | 1.8 (1.4-2.4) | **<0.001** | 9.3 | 1.4 (1.1-1.9) | **0.011** | 8.2 | 1.5 (1.1-2.0) | **0.006** | 1.7 | 1.5 (0.8-2.9) | 0.192 |
| Age at diagnosis | 0-5 years at dx | 14.0 | Ref. |  | 1.5 | Ref. |  | 2.7 | Ref. |  | 3.4 | Ref. |  | 5.0 | Ref. |  | 6.6 | Ref. |  | 1.1 | Ref. |  |
|  | 6-10 years at dx | 20.6 | 1.5 (1.2-1.8) | **<0.001** | 2.3 | 1.5 (0.8-2.9) | 0.172 | 4.5 | 1.7 (1.0-2.7) | **0.025** | 6.3 | 1.8 (1.2-2.8) | **0.002** | 6.3 | 1.3 (0.9-1.8) | 0.170 | 6.8 | 1.0 (0.7-1.4) | 0.860 | 0.7 | 0.6 (0.2-1.7) | 0.323 |
|  | 11-15 years at dx | 23.9 | 1.7 (1.4-2.1) | **<0.001** | 3.9 | 2.6 (1.5-4.5) | **<0.001** | 3.0 | 1.1 (0.7-1.8) | 0.641 | 9.8 | 2.9 (2.0-4.1) | **<0.001** | 10.6 | 2.1 (1.6-2.9) | **<0.001** | 5.9 | 0.9 (0.6-1.2) | 0.482 | 1.7 | 1.6 (0.7-3.3) | 0.209 |
|  | 16-20 years at dx | 15.8 | 1.1 (0.9-1.5) | 0.335 | 3.0 | 2.0 (1.0-3.8) | **0.034** | 2.0 | 0.8 (0.4-1.5) | 0.410 | 4.2 | 1.2 (0.7-2.0) | 0.410 | 7.9 | 1.6 (1.1-2.3) | **0.012** | 3.4 | 0.5 (0.3-0.8) | **0.003** | 1.7 | 1.6 (0.7-3.7) | 0.232 |
| Surgery | No | 16.2 | Ref. |  | 3.3 | Ref. |  | 1.4 | Ref. |  | 4.3 | Ref. |  | 7.4 | Ref. |  | 4.0 | Ref. |  | 0.5 | Ref. |  |
|  | Yes | 19.7 | 1.2 (1.0-1.4) | **0.027** | 2.3 | 0.7 (0.5-1.1) | 0.085 | 3.9 | 2.7 (1.6-4.8) | **<0.001** | 6.7 | 1.5 (1.1-2.1) | **0.004** | 7.0 | 0.9 (0.7-1.2) | 0.648 | 7.0 | 1.8 (1.3-2.4) | **<0.001** | 1.5 | 2.7 (1.2-7.3) | **0.008** |
| Chemotherapy | No | 15.3 | Ref. |  | 2.3 | Ref. |  | 2.7 | Ref. |  | 4.3 | Ref. |  | 5.7 | Ref. |  | 7.3 | Ref. |  | 0.7 | Ref. |  |
|  | Yes | 19.6 | 1.3 (1.0-1.6) | **0.016** | 2.8 | 1.2 (0.7-2.2) | 0.433 | 3.2 | 1.2 (0.8-2.0) | 0.431 | 6.4 | 1.5 (1.0-2.2) | **0.028** | 7.6 | 1.3 (1.0-1.9) | 0.068 | 5.7 | 0.8 (0.6-1.1) | 0.114 | 1.3 | 1.8 (0.8-5.2) | 0.167 |
| Radiotherapy | No | 18.7 | Ref. |  | 2.7 | Ref. |  | 3.0 | Ref. |  | 6.2 | Ref. |  | 7.6 | Ref. |  | 5.3 | Ref. |  | 1.0 | Ref. |  |
|  | Yes | 18.4 | 1.0 (0.8-1.2) | 0.816 | 2.7 | 1.0 (0.6-1.5) | 0.972 | 3.1 | 1.0 (0.7-1.6) | 0.827 | 5.2 | 0.8 (0.6-1.1) | 0.242 | 6.4 | 0.8 (0.6-1.1) | 0.166 | 7.5 | 1.4 (1.1-1.9) | **0.013** | 1.6 | 1.7 (0.9-3.1) | 0.080 |
| SCT | No | 18.3 | Ref. |  | 2.6 | Ref. |  | 3.1 | Ref. |  | 5.7 | Ref. |  | 7.1 | Ref. |  | 6.0 | Ref. |  | 1.1 | Ref. |  |
|  | Yes | 27.6 | 1.5 (1.0-2.2) | **0.044** | 5.2 | 2.0 (0.7-4.5) | 0.133 | 2.6 | 0.8 (0.2-2.5) | 0.816 | 9.7 | 1.7 (0.8-3.1) | 0.107 | 8.8 | 1.2 (0.6-2.3) | 0.495 | 8.0 | 1.3 (0.6-2.6) | 0.398 | 4.3 | 3.9 (1.2-9.8) | **0.016** |
| Year of diagnosis | 1970-1980 | 10.6 | Ref. |  | 1.3 | Ref. |  | 2.1 | Ref. |  | 2.7 | Ref. |  | 3.6 | Ref. |  | 4.8 | Ref. |  | 0.9 | Ref. |  |
|  | 1981-1985 | 12.1 | 1.1 (0.8-1.7) | 0.470 | 1.4 | 1.1 (0.4-3.3) | 0.920 | 2.4 | 1.2 (0.5-2.7) | 0.716 | 3.1 | 1.2 (0.6-2.4) | 0.677 | 5.1 | 1.4 (0.8-2.6) | 0.205 | 5.1 | 1.1 (0.6-1.9) | 0.813 | 0.6 | 0.6 (0.1-2.7) | 0.446 |
|  | 1986-1990 | 15.4 | 1.5 (1.0-2.1) | **0.026** | 2.4 | 1.9 (0.8-5.3) | 0.147 | 4.1 | 1.9 (1.0-4.3) | 0.050 | 4.7 | 1.7 (0.9-3.5) | 0.077 | 6.1 | 1.7 (1.0-3.0) | **0.047** | 5.9 | 1.2 (0.7-2.1) | 0.413 | 0.8 | 0.9 (0.2-3.6) | 0.847 |
|  | 1991-1995 | 15.8 | 1.5 (1.1-2.1) | **0.017** | 2.2 | 1.7 (0.7-4.8) | 0.241 | 2.1 | 1.0 (0.4-2.4) | 0.993 | 4.1 | 1.5 (0.8-3.1) | 0.190 | 5.7 | 1.6 (0.9-2.9) | 0.083 | 4.9 | 1.0 (0.6-1.8) | 0.949 | 1.1 | 1.2 (0.3-4.4) | 0.809 |
|  | 1996-2000 | 25.1 | 2.4 (1.7-3.4) | **<0.001** | 3.7 | 2.9 (1.1-8.0) | **0.014** | 4.5 | 2.2 (1.0-4.9) | **0.031** | 8.7 | 3.2 (1.7-6.4) | **<0.001** | 9.5 | 2.6 (1.5-4.8) | **<0.001** | 7.3 | 1.5 (0.9-2.7) | 0.106 | 1.7 | 1.9 (0.6-7.0) | 0.277 |
|  | 2001-2005 | 25.6 | 2.4 (1.6-3.6) | **<0.001** | 3.6 | 2.7 (0.9-8.5) | **0.043** | 2.8 | 1.4 (0.5-3.7) | 0.504 | 10.0 | 3.7 (1.9-7.7) | **<0.001** | 8.4 | 2.3 (1.2-4.5) | **0.007** | 7.0 | 1.5 (0.8-2.8) | 0.224 | 2.5 | 2.6 (0.7-10.6) | 0.104 |
|  | 2006-2010 | 36.6 | 3.5 (2.3-5.2) | **<0.001** | 5.4 | 4.2 (1.4-12.9) | **0.004** | 3.8 | 1.8 (0.6-5.1) | 0.229 | 12.5 | 4.6 (2.3-9.8) | **<0.001** | 18.7 | 5.2 (2.9-9.7) | **<0.001** | 8.9 | 1.8 (0.9-3.6) | 0.064 | 1.1 | 1.1 (0.1-6.9) | 0.846 |
|  | 2011-2015 | 41.9 | 4.0 (2.7-5.9) | **<0.001** | 5.2 | 4.0 (1.3-12.6) | **0.008** | 3.4 | 1.7 (0.5-4.9) | 0.333 | 16.7 | 6.2 (3.1-12.7) | **<0.001** | 17.3 | 4.8 (2.6-9.1) | **<0.001** | 8.9 | 1.8 (0.9-3.7) | 0.071 | 4.0 | 4.3 (1.2-17.2) | **0.015** |
| Age at study | 15-24 years | 25.1 | Ref. |  | 3.4 | Ref. |  | 4.0 | Ref. |  | 8.8 | Ref. |  | 9.1 | Ref. |  | 8.1 | Ref. |  | 1.6 | Ref. |  |
|  | 25-34 years | 13.9 | 0.6 (0.5-0.7) | **<0.001** | 1.6 | 0.5 (0.3-0.8) | **0.002** | 2.4 | 0.6 (0.4-0.9) | **0.021** | 4.0 | 0.5 (0.3-0.6) | **<0.001** | 5.7 | 0.6 (0.5-0.8) | **0.001** | 5.0 | 0.6 (0.5-0.8) | **0.001** | 0.9 | 0.5 (0.3-1.1) | 0.069 |
|  | 35-44 years | 11.9 | 0.5 (0.4-0.6) | **<0.001** | 2.0 | 0.6 (0.3-1.1) | 0.060 | 2.3 | 0.6 (0.3-1.0) | **0.048** | 3.0 | 0.3 (0.2-0.5) | **<0.001** | 5.6 | 0.6 (0.4-0.9) | **0.005** | 3.3 | 0.4 (0.3-0.6) | **<0.001** | 1.1 | 0.7 (0.3-1.6) | 0.377 |
|  | 45-54 years | 14.9 | 0.6 (0.4-0.9) | **0.005** | 4.0 | 1.2 (0.5-2.4) | 0.619 | 2.7 | 0.7 (0.2-1.5) | 0.363 | 4.1 | 0.5 (0.2-0.9) | **0.016** | 6.3 | 0.7 (0.4-1.2) | 0.191 | 4.3 | 0.5 (0.2-1.0) | **0.049** | 0.9 | 0.5 (0.1-2.1) | 0.420 |
|  | 55-64 years | 21.0 | 0.8 (0.1-3.0) | 0.883 | 0.0 | 0.0 (0.0-10.0) | 0.684 | 0.0 | 0.0 (0.0-8.6) | 0.644 | 0.0 | 0.0 (0.0-3.8) | 0.376 | 9.0 | 1.0 (0.0-5.6) | 0.908 | 10.5 | 1.3 (0.0-7.4) | 0.720 | 0.0 | 0.0 (0.0-21.8) | 0.835 |

Note: Bold font indicates statistically significant difference as compared to the reference group at p<0.05; Abbreviations: CI=confidence interval, CNS=central nervous system, Ref.=reference category, SCT=stem cell therapy

Supplemental Table 6a. Number of missings in outcomes of Table 2 before and after imputation (imputation - sensitivity analysis 3 “worst case”).

|  | Prevalence | | Cumulative Incidence | | Condition no longer prevalent^a^ | | Age at incidence^b^ | | Time since diagnosis at incidence^c^ | |
| --- | --- | --- | --- | --- | --- | --- | --- | --- | --- | --- |
|  | Before n= (%) | After n= (%) | Before n= (%) | After n= (%) | Before n= (%) | After n= (%) | Before n= (%) | After n= (%) | Before n= (%) | After n= (%) |
| Any musculoskeletal health condition | 129 (4.9) | 129 (4.9) | 190 (7.2) | 129 (4.9) | n.a. | n.a. | 125 (19.4) | 0 (0.0) | 125 (19.4) | 0 (0.0) |
| Osteoporosis | 129 (4.9) | 129 (4.9) | 130 (4.9) | 129 (4.9) | n.a. | n.a. | 19 (18.4) | 0 (0.0) | 19 (18.4) | 0 (0.0) |
| Arm- or leg-length discrepancy | 129 (4.9) | 129 (4.9) | 142 (5.4) | 129 (4.9) | n.a. | n.a. | 33 (27.3) | 0 (0.0) | 33 (27.3) | 0 (0.0) |
| Limited joint mobility | 129 (4.9) | 129 (4.9) | 135 (5.1) | 129 (4.9) | n.a. | n.a. | 36 (16.0) | 0 (0.0) | 36 (16.0) | 0 (0.0) |
| Persistent pain in bones or joints | 129 (4.9) | 129 (4.9) | 132 (5.0) | 129 (4.9) | n.a. | n.a. | 50 (17.9) | 0 (0.0) | 50 (17.9) | 0 (0.0) |
| Scoliosis | 129 (4.9) | 129 (4.9) | 166 (6.3) | 129 (4.9) | n.a. | n.a. | 73 (31.7) | 0 (0.0) | 73 (31.7) | 0 (0.0) |
| Changes to chest/ribs | 129 (4.9) | 129 (4.9) | 135 (5.1) | 129 (4.9) | n.a. | n.a. | 14 (28.0) | 0 (0.0) | 14 (28.0) | 0 (0.0) |

Abbreviations: n.a.=not applicable

^a^ Condition no longer prevalent: Number of survivors that indicated cumulative incidence 'yes' and prevalence 'no'. See missings listed under 'Prevalence' and 'Cumulative Incidence'.

^b^ Missing values in 'age at incidence' are only displayed for survivors who had cumulative incidence: yes (see **Table 2**)

^c^ Missing values in 'time since diagnosis at incidence' are only displayed for survivors who had cumulative incidence: yes (see **Table 2**)

Supplemental Table 6b. Prevalence and incidence of musculoskeletal health conditions in adolescent and adult 5-year survivors of childhood and adolescent cancer (N=2645; imputation - sensitivity analysis 3 “worst case”).

|  | Prevalence | | Cumulative Incidence | | Condition no longer prevalent | | Age at incidence | | Time since diagnosis  at incidence^a^ | |
| --- | --- | --- | --- | --- | --- | --- | --- | --- | --- | --- |
|  |  | |  | |  | | in years | | in years | |
|  | Before n (%) | After n= (%) | Before n (%) | After n= (%) | Before  N=^b^ (%^c^) | After  N=^b^ (%^c^) | Before  Mean  (SD; range) | After  Mean  (SD; range) | Before  Mean  (SD; range) | After  Mean  (SD; range) |
| Any MSHC | 533 (21.2) | 533 (21.2) | 643 (26.2) | 643 (25.6) | 154 (24.0) | 154 (24.0) | 16.8 (8.47; 0-56) | 16.6 (8.28; 0-56) | 7.3 (7.53; 0-38) | 7.1 (7.16; 0-38) |
| Osteoporosis | 61 (2.4) | 61 (2.4) | 103 (4.1) | 103 (4.1) | 42 (40.8) | 42 (40.8) | 19.2 (9.62; 2-45) | 19.0 (9.22; 2-45) | 8.1 (8.20; 0-30) | 8.4 (8.01; 0-30) |
| Arm- or leg-length discrepancy | 107 (4.3) | 107 (4.3) | 121 (4.8) | 121 (4.8) | 23 (19.0) | 23 (19.0) | 14.1 (8.09; 0-43) | 13.3 (7.89; 0-43) | 5.2 (6.60; 0-32) | 5.1 (6.36; 0-32) |
| Limited joint mobility | 170 (6.8) | 170 (6.8) | 225 (9.0) | 225 (8.9) | 60 (26.7) | 60 (26.7) | 15.3 (6.96; 0-43) | 15.5 (7.00; 0-43) | 5.3 (6.55; 0-32) | 5.3 (6.16; 0-32) |
| Persistent pain in bones or joints | 188 (7.5) | 188 (7.5) | 280 (11.1) | 280 (11.1) | 93 (33.2) | 93 (33.2) | 20.0 (9.16; 1-56) | 20.2 (8.88; 1-56) | 9.8 (8.77; 0-38) | 9.9 (8.45; 0-38) |
| Scoliosis | 207 (8.2) | 207 (8.2) | 230 (9.3) | 230 (9.1) | 49 (21.3) | 49 (21.3) | 14.3 (7.06; 0-44) | 14.3 (6.81; 0-44) | 6.8 (6.37; 0-29) | 6.4 (5.52; 0-29) |
| Changes to chest/ribs | 41 (1.6) | 41 (1.6) | 50 (2.0) | 50 (2.0) | 13 (26.0) | 13 (26.0) | 17.0 (9.18; 6-50) | 16.3 (8.61; 3-50) | 6.1 (7.34; 0-33) | 6.1 (6.71; 0-33) |

Note: Proportions were calculated excluding missing values. Abbreviations: MSHC=musculoskeletal health conditions, n.a.=not applicable, SD=standard deviation
^a^ **Supplemental Figure 1** displays years since diagnosis at incidence for the specific musculoskeletal health condition

^b^ number of survivors that indicated cumulative incidence 'yes' and prevalence 'no'

^c^ number of survivors that indicated cumulative incidence 'yes' and prevalence 'no' divided by number of survivors that indicated cumulative incidence 'yes'

Supplemental Table 6c. Incidence rates (per 1000 person-years) and rate ratios of musculoskeletal health conditions in adolescent and adult 5-year survivors of childhood and adolescent cancer (N=2645; imputation - sensitivity analysis 3 “worst case”).

|  | | Any musculoskeletal health condition | | | Osteoporosis | |  | Arm- or leg-length discrepancy | | | Limited joint mobility | | | Persistent pain in bones or joints | | | Scoliosis |  |  | Changes to chest/ribs | | |
| --- | --- | --- | --- | --- | --- | --- | --- | --- | --- | --- | --- | --- | --- | --- | --- | --- | --- | --- | --- | --- | --- | --- |
|  |  | Incidence rate | Rate ratios (95% CI)a | p-value | Incidence rate | Rate ratios (95% CI)a | p-value | Incidence rate | Rate ratios (95% CI)a | p-value | Incidence rate | Rate ratios (95% CI)a | p-value | Incidence rate | Rate ratios (95% CI)a | p-value | Incidence rate | Rate ratios (95% CI)a | p-value | Incidence rate | Rate ratios (95% CI)a | p-value |
|  | Overall (95% CI) | 18.4 (17.0-19.8) | n.a. |  | 2.6 (2.1-3.1) | n.a. |  | 3.1 (2.6-3.6) | n.a. |  | 5.8 (5.1-6.6) | n.a. |  | 7.1 (6.3-8.0) | n.a. |  | 6.0 (5.2-6.8) | n.a. |  | 1.2 (0.9-1.6) | n.a. |  |
| Sex | Males | 15.5 | Ref. |  | 1.7 | Ref. |  | 2.5 | Ref. |  | 5.2 | Ref. |  | 5.8 | Ref. |  | 5.1 | Ref. |  | 1.4 | Ref. |  |
|  | Females | 21.8 | 1.4 (1.2-1.6) | **<0.001** | 3.5 | 2.0 (1.3-3.1) | **0.001** | 3.7 | 1.5 (1.0-2.2) | **0.029** | 6.4 | 1.2 (0.9-1.6) | 0.132 | 8.7 | 1.5 (1.2-1.9) | **0.001** | 7.0 | 1.4 (1.1-1.8) | **0.015** | 1.0 | 0.7 (0.4-1.3) | 0.284 |
| Diagnosis | Leukaemia | 16.8 | Ref. |  | 3.3 | Ref. |  | 1.6 | Ref. |  | 4.8 | Ref. |  | 7.7 | Ref. |  | 3.9 | Ref. |  | 0.5 | Ref. |  |
|  | Lymphoma | 15.5 | 0.9 (0.7-1.2) | 0.501 | 1.7 | 0.5 (0.3-1.0) | **0.025** | 1.2 | 0.8 (0.3-1.7) | 0.473 | 3.3 | 0.7 (0.4-1.1) | 0.091 | 5.9 | 0.8 (0.5-1.1) | 0.144 | 4.3 | 1.1 (0.7-1.8) | 0.657 | 2.2 | 4.4 (1.7-13.6) | **0.001** |
|  | CNS tumour | 18.8 | 1.1 (0.9-1.4) | 0.362 | 3.8 | 1.2 (0.7-2.0) | 0.566 | 2.9 | 1.9 (0.9-3.8) | 0.069 | 6.3 | 1.3 (0.8-2.0) | 0.206 | 5.9 | 0.8 (0.5-1.1) | 0.173 | 8.2 | 2.1 (1.4-3.2) | **0.001** | 1.5 | 3.1 (1.0-10.5) | **0.035** |
|  | Neuroblastoma | 17.0 | 1.0 (0.6-1.6) | 0.928 | 1.2 | 0.4 (0.0-1.4) | 0.149 | 3.8 | 2.4 (0.8-6.2) | 0.083 | 3.8 | 0.8 (0.3-1.8) | 0.617 | 5.2 | 0.7 (0.3-1.4) | 0.279 | 12.6 | 3.2 (1.8-5.7) | **<0.001** | 0.6 | 1.2 (0.0-10.2) | 0.782 |
|  | Retinoblastoma | 10.4 | 0.6 (0.3-1.2) | 0.173 | 0.0 | 0.0 (0.0-1.4) | 0.069 | 2.4 | 1.5 (0.2-6.3) | 0.554 | 3.7 | 0.8 (0.2-2.4) | 0.725 | 6.2 | 0.8 (0.3-2.0) | 0.682 | 5.0 | 1.3 (0.3-3.5) | 0.618 | 0.0 | 0.0 (0.0-12.2) | 0.668 |
|  | Renal tumour | 16.8 | 1.0 (0.7-1.5) | 0.983 | 0.9 | 0.3 (0.0-1.1) | 0.053 | 3.9 | 2.5 (0.9-6.0) | **0.044** | 2.9 | 0.6 (0.2-1.4) | 0.243 | 3.8 | 0.5 (0.2-1.0) | **0.041** | 9.4 | 2.4 (1.3-4.2) | **0.003** | 1.4 | 2.9 (0.5-13.6) | 0.165 |
|  | Hepatic tumour | 7.9 | 0.5 (0.1-1.7) | 0.285 | 0.0 | 0.0 (0.0-4.5) | 0.430 | 0.0 | 0.0 (0.0-9.8) | 0.664 | 3.9 | 0.8 (0.0-4.7) | 0.933 | 3.9 | 0.5 (0.0-2.9) | 0.559 | 0.0 | 0.0 (0.0-3.8) | 0.364 | 0.0 | 0.0 (0.0-39.7) | 0.881 |
|  | Malignant bone tumour | 83.9 | 5.0 (3.8-6.5) | **<0.001** | 2.5 | 0.8 (0.2-2.0) | 0.637 | 28.4 | 17.9 (10.2-32.6) | **<0.001** | 47.3 | 9.8 (6.8-14.3) | **<0.001** | 25.4 | 3.3 (2.2-4.8) | **<0.001** | 12.7 | 3.2 (1.9-5.5) | **<0.001** | 2.6 | 5.2 (1.3-20.6) | **0.013** |
|  | Soft tissue sarcoma | 19.9 | 1.2 (0.8-1.7) | 0.308 | 1.1 | 0.3 (0.1-1.1) | **0.049** | 3.9 | 2.5 (1.0-5.6) | **0.029** | 7.0 | 1.4 (0.8-2.5) | 0.191 | 5.0 | 0.6 (0.3-1.2) | 0.135 | 7.6 | 2.0 (1.1-3.4) | **0.019** | 1.5 | 3.1 (0.6-13.0) | 0.105 |
|  | Germ cell tumour | 10.8 | 0.6 (0.4-1.0) | **0.039** | 3.1 | 1.0 (0.4-2.2) | 0.941 | 0.9 | 0.5 (0.1-2.3) | 0.445 | 1.3 | 0.3 (0.1-0.8) | **0.010** | 4.9 | 0.6 (0.3-1.2) | 0.138 | 2.7 | 0.7 (0.2-1.6) | 0.385 | 0.9 | 1.8 (0.2-9.8) | 0.496 |
|  | Other tumour | 17.1 | 1.0 (0.6-1.7) | 0.904 | 4.7 | 1.5 (0.4-3.7) | 0.425 | 2.8 | 1.7 (0.3-5.9) | 0.376 | 1.9 | 0.4 (0.0-1.5) | 0.157 | 5.6 | 0.7 (0.3-1.7) | 0.476 | 6.8 | 1.7 (0.7-3.9) | 0.190 | 0.9 | 1.9 (0.0-15.3) | 0.560 |
|  | Langerhans cell histiocytosis | 16.2 | 1.0 (0.6-1.6) | 0.913 | 3.0 | 0.9 (0.2-2.5) | 0.906 | 1.5 | 0.9 (0.1-3.9) | 0.995 | 3.7 | 0.8 (0.2-1.9) | 0.621 | 10.9 | 1.4 (0.7-2.5) | 0.230 | 7.8 | 2.0 (0.9-4.0) | 0.066 | 0.7 | 1.5 (0.0-12.5) | 0.667 |
| Relapse | No | 16.8 | Ref. |  | 2.2 | Ref. |  | 3.0 | Ref. |  | 4.9 | Ref. |  | 6.6 | Ref. |  | 5.4 | Ref. |  | 1.1 | Ref. |  |
|  | Yes | 24.8 | 1.5 (1.2-1.8) | **<0.001** | 3.8 | 1.7 (1.1-2.6) | **0.017** | 3.1 | 1.0 (0.6-1.6) | 0.935 | 9.1 | 1.8 (1.4-2.5) | **<0.001** | 9.4 | 1.4 (1.1-1.9) | **0.011** | 8.3 | 1.5 (1.1-2.1) | **0.005** | 1.7 | 1.5 (0.8-2.9) | 0.191 |
| Age at diagnosis | 0-5 years at dx | 14.2 | Ref. |  | 1.5 | Ref. |  | 2.7 | Ref. |  | 3.4 | Ref. |  | 5.0 | Ref. |  | 6.7 | Ref. |  | 1.1 | Ref. |  |
|  | 6-10 years at dx | 20.8 | 1.5 (1.2-1.8) | **<0.001** | 2.3 | 1.5 (0.8-2.9) | 0.172 | 4.5 | 1.7 (1.0-2.7) | **0.027** | 6.4 | 1.9 (1.2-2.8) | **0.002** | 6.4 | 1.3 (0.9-1.8) | 0.170 | 6.8 | 1.0 (0.7-1.4) | 0.876 | 0.7 | 0.6 (0.2-1.7) | 0.319 |
|  | 11-15 years at dx | 24.2 | 1.7 (1.4-2.1) | **<0.001** | 4.0 | 2.6 (1.5-4.5) | **<0.001** | 3.0 | 1.1 (0.7-1.8) | 0.659 | 9.8 | 2.9 (2.0-4.1) | **<0.001** | 10.6 | 2.1 (1.6-2.9) | **<0.001** | 5.9 | 0.9 (0.6-1.2) | 0.450 | 1.7 | 1.6 (0.7-3.3) | 0.211 |
|  | 16-20 years at dx | 15.9 | 1.1 (0.9-1.4) | 0.384 | 3.0 | 2.0 (1.0-3.8) | **0.034** | 2.0 | 0.8 (0.4-1.5) | 0.402 | 4.2 | 1.2 (0.7-2.0) | 0.407 | 8.0 | 1.6 (1.1-2.3) | **0.012** | 3.4 | 0.5 (0.3-0.8) | **0.003** | 1.7 | 1.6 (0.7-3.7) | 0.234 |
| Surgery | No | 16.5 | Ref. |  | 3.3 | Ref. |  | 1.4 | Ref. |  | 4.3 | Ref. |  | 7.5 | Ref. |  | 4.0 | Ref. |  | 0.5 | Ref. |  |
|  | Yes | 19.9 | 1.2 (1.0-1.4) | **0.029** | 2.4 | 0.7 (0.5-1.1) | 0.084 | 3.9 | 2.7 (1.6-4.8) | **<0.001** | 6.7 | 1.6 (1.1-2.2) | **0.004** | 7.1 | 0.9 (0.7-1.2) | 0.647 | 7.0 | 1.7 (1.3-2.4) | **<0.001** | 1.5 | 2.7 (1.2-7.3) | **0.007** |
| Chemotherapy | No | 15.4 | Ref. |  | 2.3 | Ref. |  | 2.7 | Ref. |  | 4.4 | Ref. |  | 5.7 | Ref. |  | 7.3 | Ref. |  | 0.7 | Ref. |  |
|  | Yes | 19.9 | 1.3 (1.0-1.6) | **0.015** | 2.8 | 1.2 (0.7-2.2) | 0.435 | 3.2 | 1.2 (0.7-2.0) | 0.435 | 6.5 | 1.5 (1.0-2.2) | **0.029** | 7.7 | 1.3 (1.0-1.9) | 0.070 | 5.7 | 0.8 (0.6-1.1) | 0.120 | 1.3 | 1.8 (0.8-5.2) | 0.164 |
| Radiotherapy | No | 18.9 | Ref. |  | 2.7 | Ref. |  | 3.0 | Ref. |  | 6.2 | Ref. |  | 7.6 | Ref. |  | 5.3 | Ref. |  | 1.0 | Ref. |  |
|  | Yes | 18.6 | 1.0 (0.8-1.2) | 0.846 | 2.7 | 1.0 (0.6-1.5) | 0.973 | 3.1 | 1.0 (0.7-1.6) | 0.823 | 5.3 | 0.8 (0.6-1.1) | 0.255 | 6.4 | 0.8 (0.6-1.1) | 0.167 | 7.5 | 1.4 (1.1-1.9) | **0.013** | 1.6 | 1.7 (0.9-3.2) | 0.078 |
| SCT | No | 18.6 | Ref. |  | 2.6 | Ref. |  | 3.1 | Ref. |  | 5.8 | Ref. |  | 7.1 | Ref. |  | 6.0 | Ref. |  | 1.1 | Ref. |  |
|  | Yes | 27.6 | 1.5 (1.0-2.2) | 0.051 | 5.2 | 2.0 (0.7-4.5) | 0.132 | 2.6 | 0.8 (0.2-2.5) | 0.810 | 9.8 | 1.7 (0.8-3.1) | 0.109 | 8.8 | 1.2 (0.6-2.3) | 0.493 | 7.9 | 1.3 (0.6-2.5) | 0.417 | 4.3 | 3.9 (1.2-9.7) | **0.016** |
| Year of diagnosis | 1970-1980 | 10.9 | Ref. |  | 1.3 | Ref. |  | 2.1 | Ref. |  | 2.7 | Ref. |  | 3.6 | Ref. |  | 4.9 | Ref. |  | 0.9 | Ref. |  |
|  | 1981-1985 | 12.4 | 1.1 (0.8-1.7) | 0.461 | 1.4 | 1.1 (0.4-3.3) | 0.921 | 2.4 | 1.2 (0.5-2.8) | 0.702 | 3.1 | 1.1 (0.6-2.4) | 0.699 | 5.2 | 1.4 (0.8-2.7) | 0.197 | 5.2 | 1.1 (0.6-1.9) | 0.815 | 0.6 | 0.6 (0.1-2.7) | 0.447 |
|  | 1986-1990 | 15.7 | 1.4 (1.0-2.1) | **0.030** | 2.5 | 1.9 (0.8-5.3) | 0.146 | 4.1 | 1.9 (1.0-4.3) | **0.050** | 4.7 | 1.7 (0.9-3.4) | 0.081 | 6.1 | 1.7 (1.0-3.1) | **0.044** | 6.0 | 1.2 (0.7-2.1) | 0.438 | 0.8 | 0.9 (0.2-3.6) | 0.844 |
|  | 1991-1995 | 15.8 | 1.5 (1.0-2.1) | **0.025** | 2.2 | 1.7 (0.7-4.8) | 0.240 | 2.1 | 1.0 (0.4-2.4) | 0.996 | 4.1 | 1.5 (0.8-3.0) | 0.206 | 5.7 | 1.6 (0.9-2.9) | 0.082 | 4.9 | 1.0 (0.6-1.7) | 0.992 | 1.1 | 1.2 (0.3-4.4) | 0.817 |
|  | 1996-2000 | 25.1 | 2.3 (1.6-3.3) | **<0.001** | 3.7 | 2.9 (1.2-8.1) | **0.013** | 4.5 | 2.2 (1.0-4.9) | **0.032** | 8.8 | 3.2 (1.7-6.3) | **<0.001** | 9.5 | 2.6 (1.5-4.8) | **<0.001** | 7.3 | 1.5 (0.9-2.6) | 0.127 | 1.7 | 1.8 (0.6-7.0) | 0.281 |
|  | 2001-2005 | 25.5 | 2.3 (1.6-3.5) | **<0.001** | 3.6 | 2.7 (0.9-8.5) | **0.043** | 2.8 | 1.4 (0.5-3.7) | 0.510 | 10.0 | 3.7 (1.9-7.5) | **<0.001** | 8.4 | 2.3 (1.2-4.5) | **0.007** | 6.9 | 1.4 (0.7-2.7) | 0.257 | 2.5 | 2.6 (0.7-10.5) | 0.106 |
|  | 2006-2010 | 35.8 | 3.3 (2.2-4.9) | **<0.001** | 5.4 | 4.2 (1.4-12.9) | **0.004** | 3.8 | 1.8 (0.6-5.1) | 0.231 | 12.4 | 4.5 (2.2-9.5) | **<0.001** | 18.6 | 5.2 (2.9-9.7) | **<0.001** | 8.6 | 1.8 (0.9-3.5) | 0.086 | 1.1 | 1.1 (0.1-6.9) | 0.850 |
|  | 2011-2015 | 41.5 | 3.8 (2.6-5.7) | **<0.001** | 5.2 | 4.0 (1.3-12.6) | **0.008** | 3.4 | 1.6 (0.5-4.9) | 0.336 | 16.6 | 6.0 (3.1-12.5) | **<0.001** | 17.2 | 4.8 (2.6-9.1) | **<0.001** | 8.8 | 1.8 (0.9-3.6) | 0.081 | 4.0 | 4.3 (1.2-17.0) | **0.016** |
| Age at study | 15-24 years | 25.1 | Ref. |  | 3.4 | Ref. |  | 4.0 | Ref. |  | 8.8 | Ref. |  | 9.1 | Ref. |  | 8.1 | Ref. |  | 1.6 | Ref. |  |
|  | 25-34 years | 14.2 | 0.6 (0.5-0.7) | **<0.001** | 1.6 | 0.5 (0.3-0.8) | **0.002** | 2.5 | 0.6 (0.4-1.0) | **0.022** | 4.0 | 0.5 (0.3-0.6) | **<0.001** | 5.7 | 0.6 (0.5-0.8) | **0.001** | 5.1 | 0.6 (0.5-0.8) | **0.002** | 0.9 | 0.5 (0.3-1.1) | 0.071 |
|  | 35-44 years | 12.1 | 0.5 (0.4-0.6) | **<0.001** | 2.0 | 0.6 (0.3-1.1) | 0.060 | 2.3 | 0.6 (0.3-1.0) | **0.048** | 3.1 | 0.3 (0.2-0.6) | **<0.001** | 5.6 | 0.6 (0.4-0.9) | **0.005** | 3.4 | 0.4 (0.3-0.7) | **<0.001** | 1.1 | 0.7 (0.3-1.6) | 0.384 |
|  | 45-54 years | 15.3 | 0.6 (0.4-0.9) | **0.008** | 4.0 | 1.2 (0.5-2.4) | 0.622 | 2.7 | 0.7 (0.2-1.6) | 0.373 | 4.2 | 0.5 (0.2-0.9) | **0.018** | 6.4 | 0.7 (0.4-1.2) | 0.202 | 4.3 | 0.5 (0.2-1.0) | 0.053 | 0.9 | 0.5 (0.1-2.1) | 0.420 |
|  | 55-64 years | 24.3 | 1.0 (0.1-3.5) | 0.956 | 0.0 | 0.0 (0.0-10.0) | 0.683 | 0.0 | 0.0 (0.0-8.6) | 0.644 | 0.0 | 0.0 (0.0-3.8) | 0.376 | 9.0 | 1.0 (0.0-5.6) | 0.910 | 12.1 | 1.5 (0.0-8.5) | 0.631 | 0.0 | 0.0 (0.0-21.8) | 0.835 |

Note: Bold font indicates statistically significant difference as compared to the reference group at p<0.05; Abbreviations: CI=confidence interval, CNS=central nervous system, Ref.=reference category, SCT=stem cell therapy
